# Antigenic Characterization of New Lineage II Insect-Specific Flaviviruses in Australian Mosquitoes and Identification of Host Restriction Factors

**DOI:** 10.1128/mSphere.00095-20

**Published:** 2020-06-17

**Authors:** Jessica J. Harrison, Jody Hobson-Peters, Agathe M. G. Colmant, Joanna Koh, Natalee D. Newton, David Warrilow, Helle Bielefeldt-Ohmann, Thisun B. H. Piyasena, Caitlin A. O’Brien, Laura J. Vet, Devina Paramitha, James R. Potter, Steven S. Davis, Cheryl A. Johansen, Yin Xiang Setoh, Alexander A. Khromykh, Roy A. Hall

**Affiliations:** a School of Chemistry and Molecular Biosciences, The University of Queensland, St. Lucia, Queensland, Australia; b Australian Infectious Diseases Research Centre, The University of Queensland, St. Lucia, Queensland, Australia; c Public Health Virology Laboratory, Department of Health, Queensland Government, Archerfield, Queensland, Australia; d Berrimah Veterinary Laboratory, Department of Primary Industries and Fisheries, Darwin, Australia; e School of Pathology and Laboratory Medicine, WA, Nedlands, Western Australia, Australia; f PathWest Laboratory Medicine WA, Nedlands, Western Australia, Australia; University of Chicago

**Keywords:** *Aedeomyia catasticta*, *Aedes normanensis*, Binjari virus, Hidden Valley virus, chimeric virus, circular polymerase extension reaction, host restriction, insect-specific flavivirus, lineage II insect-specific flavivirus, monoclonal antibodies

## Abstract

We describe two new insect-specific flaviviruses (ISFs) isolated from mosquitoes in Australia, Binjari virus (BinJV) and Hidden Valley virus (HVV), that grow efficiently in mosquito cells but fail to replicate in a range of vertebrate cell lines. Phylogenetic analysis revealed that BinJV and HVV were closely related (90% amino acid sequence identity) and clustered with lineage II (dual-host affiliated) ISFs, including the Lammi and Nounané viruses. Using a panel of monoclonal antibodies prepared to BinJV viral proteins, we confirmed a close relationship between HVV and BinJV and revealed that they were antigenically quite divergent from other lineage II ISFs. We also constructed chimeric viruses between BinJV and the vertebrate-infecting West Nile virus (WNV) by swapping the structural genes (prM and E) to produce BinJ/WNV_KUN_-prME and WNV_KUN_/BinJV-prME. This allowed us to assess the role of different regions of the BinJV genome in vertebrate host restriction and revealed that while BinJV structural proteins facilitated entry to vertebrate cells, the process was inefficient. In contrast, the BinJV replicative components in wild-type BinJV and BinJ/WNV_KUN_-prME failed to initiate replication in a wide range of vertebrate cell lines at 37°C, including cells lacking components of the innate immune response. However, trace levels of replication of BinJ/WNV_KUN_-prME could be detected in some cultures of mouse embryo fibroblasts (MEFs) deficient in antiviral responses (IFNAR^−/−^ MEFs or RNase L^−/−^ MEFs) incubated at 34°C after inoculation. This suggests that BinJV replication in vertebrate cells is temperature sensitive and restricted at multiple stages of cellular infection, including inefficient cell entry and susceptibility to antiviral responses.

**IMPORTANCE** The globally important flavivirus pathogens West Nile virus, Zika virus, dengue viruses, and yellow fever virus can infect mosquito vectors and be transmitted to humans and other vertebrate species in which they cause significant levels of disease and mortality. However, the subgroup of closely related flaviviruses, known as lineage II insect-specific flaviviruses (Lin II ISFs), only infect mosquitoes and cannot replicate in cells of vertebrate origin. Our data are the first to uncover the mechanisms that restrict the growth of Lin II ISFs in vertebrate cells and provides new insights into the evolution of these viruses and the mechanisms associated with host switching that may allow new mosquito-borne viral diseases to emerge. The new reagents generated in this study, including the first Lin II ISF-reactive monoclonal antibodies and Lin II ISF mutants and chimeric viruses, also provide new tools and approaches to enable further research advances in this field.

## Abstract (precis)

The globally important flavivirus pathogens West Nile virus, Zika virus, dengue viruses, and yellow fever virus can infect mosquito vectors and be transmitted to humans and other vertebrate species in which they cause significant levels of disease and mortality. However, the subgroup of closely related flaviviruses, known as lineage II insect-specific flaviviruses (Lin II ISFs), only infect mosquitoes and cannot replicate in cells of vertebrate origin. Our data are the first to uncover the mechanisms that restrict the growth of Lin II ISFs in vertebrate cells and provides new insights into the evolution of these viruses and the mechanisms associated with host switching that may allow new mosquito-borne viral diseases to emerge. The new reagents generated in this study, including the first Lin II ISF-reactive monoclonal antibodies and Lin II ISF mutants and chimeric viruses, also provide new tools and approaches to enable further research advances in this field.

## INTRODUCTION

The genus *Flavivirus* (Family: *Flaviviridae*) comprises more than 70 distinct members which include many important mosquito-borne pathogens, such as dengue, Japanese encephalitis, yellow fever, Zika, and West Nile viruses. Members of this genus have a positive-sense, single-stranded RNA genome approximately 11 kb in size, consisting of a large open reading frame (ORF) which encodes three structural and seven nonstructural genes, flanked by 5′ and 3′ untranslated regions (UTRs). Virions are spherical in shape and approximately 50 nm in diameter. While the majority of flavivirus infections are asymptomatic, more severe infections may result in hemorrhagic fever or viral encephalitis (1–3).

Recent studies have identified a large group of flaviviruses, which replicate only in the mosquito host and have been termed insect-specific flaviviruses (ISFs) (4, 5). Interest in these viruses has increased rapidly due to their intriguing evolutionary relationship to vertebrate-infecting flaviviruses (VIFs) and their recent application as safe recombinant platforms for vaccines and diagnostics (6). ISFs can be separated into two genetic clades; lineage I (classical) ISFs form a distinct clade that is quite divergent from VIFs, while lineage II (dual-host affiliated) ISFs display an insect-specific phenotype but cluster phylogenetically with VIFs (4, 5).

Extensive studies utilizing chimeric viruses constructed between the Australian lineage I ISF Palm Creek virus (PCV) and WNV Kunjin subtype (WNV_KUN_), as well as Niénokoué virus (NIEV) and yellow fever virus (YFV), have demonstrated that host restriction of lineage I ISFs occurs at multiple stages of cellular infection. These studies reveal that inhibition of replication occurs at the levels of viral attachment and cellular entry, genome replication, and virus assembly and release (7, 8). In contrast, and despite a rapid increase in the identification and isolation of lineage II ISFs in recent years, the precise mechanisms behind the restriction of these viruses are yet to be elucidated.

We have previously detailed the isolation and characterization of Binjari virus (BinJV), from *Aedes normanensis* mosquitoes collected at the Bradshaw Field Training Area, Northern Territory (6). BinJV is the first lineage II ISF found in Australia and was shown to be noninfectious in vertebrate cells and tolerant for exchange of its structural (prM-E) protein genes with those from a range of pathogenic flaviviruses. Here, we describe the isolation and characterization of Hidden Valley virus (HVV), a close genetic relative of BinJV, which was isolated from *Aedeomyia catasticta* mosquitoes collected at Kununurra, Western Australia. To expand on our previous studies on BinJV, we also employed circular polymerase extension reaction (CPER) to produce a panel of mutant and chimeric viruses to explore the antigenic structure of lineage II ISFs and the mechanisms of their host restriction.

## RESULTS

### Isolation, morphological, and bioinformatic analysis of Hidden Valley and Binjari viruses.

**FIG 1:**
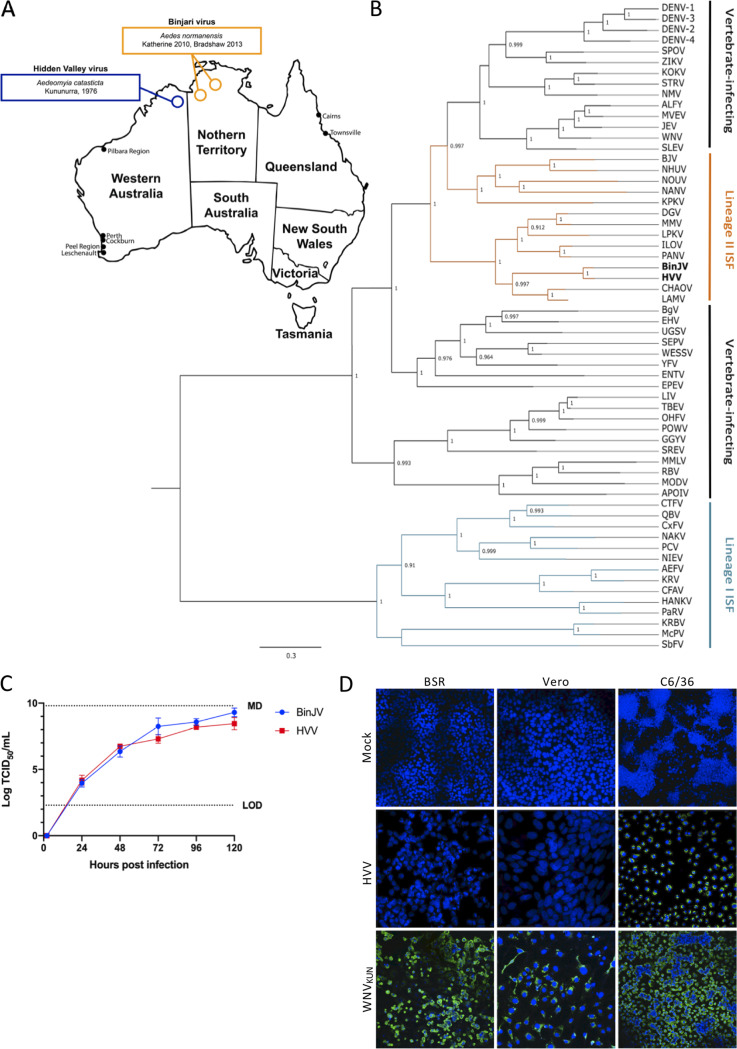
Characterization of BinJV and HVV. (A) BinJV and HVV were isolated from mosquitoes captured at the Bradshaw Field Training Area (BFTA), Northern Territory, and Kununurra, northern Western Australia, respectively. (B) Dendrogram showing phylogenetic relationship between BinJV, HVV, and other flaviviruses using a maximum-likelihood model and complete amino acid sequences. Sequences were derived using the following GenBank accession numbers: AB488408, *Aedes* flavivirus; AY898809, Alfuy virus; KU308380, Bamaga virus; MG587038, Binjari virus; KC496020, Barkedji virus; KJ741267, cell fusing agent virus; JQ308185, Chaoyang virus; AB262759, *Culex* flavivirus; HE574574, *Culex theileri* flavivirus; U88536, dengue virus serotype 1; U87411, dengue virus serotype 2; AY099336, dengue virus serotype 3; AF326825, dengue virus serotype 4; NC_027999, Ecuador Paraiso Escondido virus; DQ859060, Edge Hill virus; DQ837641, Entebbe bat virus; DQ235145, Gadgets Gully virus; NC_030401, Hanko virus; KC692067, Ilomantsi virus; M18370, Japanese encephalitis virus; AY632541, Kokobera virus; AY149905, Kamiti River virus; KY320648, Kampung Karu virus; NC_035118, Karumba virus; KC692068, Lammi virus; Y07863, Long Pine Key virus; KY290256, Louping ill virus; NC_035187, Mac Peak virus; AJ242984 and MF139576, Marisma mosquito virus; AJ242984, Modoc virus; AF161266, Murray Valley encephalitis virus; NC_030400, Nakiwogo virus; KJ210048, Nhumirim virus; JQ957875, Nienokoue virus; KC788512, New Mappon virus; EU159426 and MF139575, Nanay virus; EU159426, Nounane virus; AY193805, Omsk hemorrhagic fever virus; KT192549, Parramatta River virus; KC505248, Palm Creek virus; KY072986, Panmunjeom flavivirus; L06436, Powassan virus; FJ644291, Quang Binh virus; NC_003675, Rio Bravo virus; DQ837642, Sepik virus; DQ525916, St. Louis encephalitis virus; DQ859064, Spondweni virus; DQ235150, Saumarez Reef virus; KM225263, Stratford virus; U27495, tick-borne encephalitis virus; DQ859065, Uganda S virus; JN226796, Wesselsbron virus; KY229074, West Nile virus; X03700, Yellow fever virus; and AY632535, Zika virus. (C) Comparative growth kinetics of BinJV and HVV in C6/36 cells infected at an MOI of 0.1. The levels of infectious virus were determined by TCID_50_ over 5 days. The limit of detection (LOD) and maximum detection (MD) levels are indicated. (D) HVV or WNV_KUN_ was used to infect the vertebrate cell lines (BSR and Vero) or mosquito C6/36 cells at an MOI of 1 and fixed 5 days postinfection. IFA analysis was performed by probing with anti-flavivirus NS1 MAb 4G4 (green). Nuclei were stained with Hoechst 33342. Images were obtained at ×20 magnification. BinJV growth in vertebrate cells has previously been reported (6).

The first detection of Binjari virus (BinJV) was from RNA extracted from a single pool of *Aedes normanensis* mosquitoes collected in Katherine, Northern Territory, Australia, in 2010 (Fig. 1A; see Table S1 in the supplemental material) (6, 9). BinJV was subsequently isolated from the same mosquito species, collected at Bradshaw Field Training Area (Northern Territory, Australia) in 2013 (6). BinJV was found in only one of 232 pools of *Aedes normanensis* mosquitoes collected over the same time period and general locality and, apart from the initial detection in 2010, was not detected in a further 89 pools of the same mosquito species collected from other regions of the Northern Territory and northern areas of Western Australia. This suggests a relatively low prevalence of BinJV in *Aedes normanensis* mosquito populations (Table S1).

10.1128/mSphere.00095-20.6 TABLE S1 Summary of mosquito pools screened for presence of BinJV and HVV. Download Table S1, DOCX file, 0.02 MB. © Crown copyright 2020. 2020 Crown This content is distributed under the terms of the Creative Commons Attribution 4.0 International license.

Hidden Valley virus (HVV) was isolated from a pool of *Aedeomyia catasticta* mosquitoes trapped at Kununurra (Western Australia) in 1976 (Fig. 1A; Table S1). Retrospective analysis of another 14 pools of *Aedeomyia catasticta* mosquitoes using the primer pair FU2 and cFD3 (10) yielded an additional seven isolates of HVV, with each isolate sharing between a 92.0 and a 99.5% nucleotide identity to the prototype isolate over a 498-nucleotide region of the NS5 gene (Table 1) indicating that they are likely strains of HVV (10). The complete ORF of the prototype HVV isolate (OR1076), was elucidated (GenBank accession number MN954647) and found to encode a 3,433-amino-acid polyprotein. HVV phylogenetically groups with BinJV, within the lineage II ISF clade with ≥99% support, and further clusters with other lineage II ISFs predominantly isolated from *Aedes* mosquito species (Fig. 1B). Sequence alignments between the nucleotide and amino acid sequences of BinJV, HVV, and selected lineage II ISFs demonstrated that BinJV and HVV share 75.4 and 90.8% identity in nucleotide and amino acid sequences, respectively, and approximately 62% amino acid sequence identity to their closest relative, Chaoyang virus (CHAOV) (Table 2). These data led us to propose that BinJV and HVV should be considered separate species within the *Flavivirus* genus, based on previously established taxonomic criteria (10). Consistent with an ISF phenotype, both viruses replicated efficiently in C6/36 cells, reaching titers of 10^9.3^ 50% tissue culture infective doses (TCID_50_)/ml (BinJV) and 10^8.5^ TCID_50_/ml (HVV) by 120 h postinfection (Fig. 1C). Similar to our previous findings with BinJV (6), HVV failed to replicate in Vero or BSR mammalian cell lines that are commonly used for flavivirus isolation (Fig. 1D) (6).

**TABLE 1:** HVV isolates

Pool ID	Date collected	Pairwise bp identity (%) to prototype^*a*^
OR285	29 Nov 73	98.5
OR587	Nov/Dec 74	99.5
OR886	1 Nov 75	92.0
OR887	1 Nov 75	99.4
OR896	3 Nov 75	98.4
OR904	29 Oct 75	99.1
OR1076^*b*^	Oct/Nov 76	100
OR1082	Oct/Nov 76	99.4

a Identity calculated over a 498-bp region of the NS5 gene.

b Prototype HVV isolate.

**TABLE 2:**
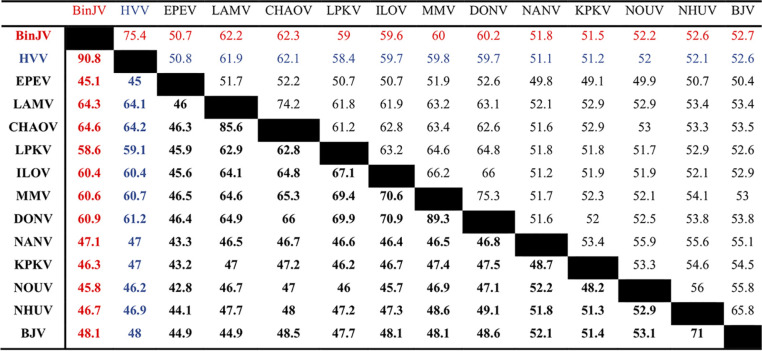
Nucleotide and amino acid sequence identities between lineage II ISF ORFs^*a*^

a Amino acid percent identities are indicated in boldface; nucleotide percent identities are indicated in regular typeface. Sequences derived based on the following accession numbers: MG587038 (BinJV; Binjari virus), MN954647 (HVV; Hidden Valley virus), KC496020 (BJV; Barkedji virus), JQ308185 (CHAOV; Chaoyang virus), NC_016997 (DONV; Donggang virus), NC_027999 (EPEV, Paraiso Escondido virus), KC692067 (ILOV; Ilomantsi virus), KY320648 (KPKV; Kampung Karu virus), KC692068 (LAMV; Lammi virus), KY290256 (LPKV; Long Pine Key virus) MF139576 (MMV; Marisma mosquito virus), MF139575 (NANV; Nanay virus), KJ210048 (NHUV; Nhumirim virus), and EU159426 (NOUV; Nounane virus).

Cleavage sites in the BinJV and HVV polyproteins were predicted according to previously published guidelines (4, 11, 12) and revealed that the ORFs code for viral proteins according to the standard flavivirus genome organization (Table 3). Consistent with studies on lineage I ISFs, such as PCV (9), several N-linked glycosylation sites were predicted within the NS1 protein, two in BinJV NS1 (positions 888 and 984) and three in HVV NS1 (positions 887, 984, and 1,084). Neither E protein was predicted to contain glycosylation sites, while a single glycosylation site was predicted for both BinJV and HVV at position 140 of prM. Transmembrane domains were predicted with TMHMM and were consistent with the classical flaviviral membrane topology, with structural (capsid and envelope) and nonstructural (NS2A, NS2B, NS4A, and NS4B) proteins containing transmembrane domains for both BinJV and HVV.

**TABLE 3:** Predicted cleavage sites

Junction	Sequence^*a*^		Cleavage
	BinJV	HVV	
C/AnchC	GKKRR ↓ GVQDV	GKNRR ↓ GLQEV	After dibasic residues
C/prM	AGAMA ↓ ATLRT	VGVFS ↓ ATLKT	Signalase-like cleavage
prM/E	APSYG ↓ NQCLD	APSYG ↓ NQCLD	Signalase-like cleavage
E/NS1	VTVGA ↓ EIGCS	VSVGA ↓ EIGCS	Signalase-like cleavage
NS1/NS2A	SHVAA ↓ GVFKG	SHVAA ↓ HVAAG	Signalase-like cleavage
NS2A/NS2B	LKGRR ↓ SWPAG	LKSRR ↓ SWPAG	After dibasic residues
NS2B/NS3	KSNKR ↓ GTVLW	KANRR ↓ GTVLW	After dibasic residues
NS3/NS4A	AEGRR ↓ RYSEL	AEGRR ↓ RYSEL	After dibasic residues
NS4A/2K	PGSQR ↓ SVQDN	PGSQR ↓ SVQDN	After dibasic residues
2K/NS4B	AIIAA ↓ NEAGL	AAIAA ↓ NEAGL	Signalase-like cleavage
NS4B/NS5	GVPRR ↓ GLQAT	GVSRR ↓ GIQAT	After dibasic residues

a Cleavage sites are indicated by “↓”.

### BinJV and HVV are antigenically distinct from VIFs and lineage I ISFs.

Antigenic analyses of lineage II ISFs remain limited to date. To assess the antigenic relationships between BinJV, HVV, and other flaviviruses and to develop crucial research tools to specifically detect lineage II ISF proteins, a panel of 21 hybridomas secreting monoclonal antibodies (MAbs) reactive to BinJV proteins were generated through hybridoma fusion technology and characterized (Table 4). The MAbs had specificities for BinJV proteins prM (25 kDa), E (50 kDa), NS1 dimer proteins (∼90 kDa), or an unidentified 75-kDa protein, presumably uncleaved prME, when assessed by Western blotting and fixed-cell enzyme-linked immunosorbent assay (ELISA) (Table 4; Fig. S1). None of the MAbs were capable of neutralizing 100 infectious units of BinJV in microneutralization assays. All MAbs were shown to be IgG isotypes, with the exception of two (BJ-4A8 and BJ-5H4) which appeared to be class switching between IgM and IgG (Table 4).

**TABLE 4:** Characterization of BinJV-reactive MAbs

MAb	Target^*a*^	Neutralization titer^*b*^	Isotype
BJ-1C1	E	<10	IgG1
BJ-1C8	E	<10	IgG1
BJ-1D2	E	<10	IgG1
BJ-1E1	E	<10	IgG1
BJ-1F2	E	<10	IgG1
BJ-1G9	prM	<10	IgG2a
BJ-2B1	prM, E	<10	IgG1
BJ-2B11	E	<10	IgG1
BJ-3A3	NS1	<10	IgG1
BJ-3E6	prM	<10	IgG3
BJ-4A8	NS1	<10	IgG1/IgM
BJ-4C1	prM	<10	IgG2a
BJ-4D10	prM, E	<10	IgG2a
BJ-5B6	prM, E, prME	<10	IgG2a
BJ-5E5	NS1	<10	IgG1
BJ-5F7	NS1	<10	IgG1
BJ-5H4	E, prME	<10	IgG1/IgM
BJ-6B11	E	<10	IgG1
BJ-6E6	E	<10	IgG1
BJ-7C4	E, prME	<10	IgG1
BJ-7G10	NS1	<10	IgG1

a Uncleaved prME = 75-kDa protein; E = 50-kDa protein; prM = 25-kDa protein. NS1 reactivity was confirmed using BinJ/WNV_KUN_-prME (expresses BinJV NS1 but not BinJV prME) in a fixed-cell ELISA (the cutoff for a positive result was 2× OD of the same MAb dilution on mock-infected cells).

b The neutralization titer was taken as the highest dilution of MAb (hybridoma culture supernatant) that inhibited virus replication.

10.1128/mSphere.00095-20.1 FIG S1 Example Western blots of BinJV reactive hybridomas. Samples were resolved by SDS-PAGE on 4 to 12% Bis-Tris gels and proteins analyzed by Western blotting with anti-BinJV mouse serum (A) or anti-BinJV hybridomas anti-E BJ-1C1 (B), anti-prM BJ-3E6 (C), anti-prM and E BJ-4D10 (D), and anti-NS1 (dimer) BJ-7G10 (E). Download FIG S1, TIF file, 0.4 MB. © Crown copyright 2020. 2020 Crown This content is distributed under the terms of the Creative Commons Attribution 4.0 International license.

When tested against HVV and other flaviviruses in ELISA, only three MAbs (two anti-prM [BJ-1G9 and BJ-4C1] and one anti-E [BJ-5H4]) failed to recognize HVV, confirming the close relationship between BinJV and HVV (Table 5). In contrast, none of the MAbs reacted to Australian lineage I ISF isolates of PCV, Parramatta River virus (PaRV), and cell fusing agent virus (CFAV). Only MAbs BJ-1E1 and BJ-6E6 recognized VIFs and were broadly cross-reactive, as evidenced by reactivity with Murray Valley encephalitis virus (MVEV), DENV-2, WNV_KUN_, Bamaga virus (BgV), and ZIKV (Table 5). Binding profiles of an extensive panel of VIF protein-targeted MAbs, including those that are broadly cross-reactive (e.g., 4G2 [13], 6B6C-1 [14], M2-1E7 [15], P3H8 [6], and 4G4 [16]), further revealed that BinJV and HVV were not recognized by most of these antibodies, demonstrating a lack of antigenic similarity between the E protein of VIFs and lineage II ISFs. Only the pan-flavivirus MAb 4G4 (raised to the NS1 protein of MVEV [16]) recognized BinJV and HVV (Table 5).

**TABLE 5:** Cross-reactivity of BinJV-derived MAbs to other flaviviruses in ELISA^*a*^

MAb	Target^*b*^	MAb reactivity in ELISA^*c*^															
		ISF										VIF					Reference
		Lineage II							Lineage I								
		BinJV	HVV	LAMV	ILOV	NHUV	CHAOV	NOUV	PaRV	PCV	CFAV	MVEV	WNV_KUN_	BgV	DENV-2	ZIKV	
BJ-1C1	E	**+**	**+**	–	–	–	**–**	**–**	–	–	–	–	–	–	–	–	6
BJ-1C8	E	**+**	**+**	–	–	–	**–**	**–**	–	–	–	–	–	–	–	–	NA
BJ-1D2	E	**+**	**+**	–	–	–	ND	ND	–	–	–	–	–	–	–	–	NA
BJ-1E1	E	**+**	**+**	**+**	**+**	**+**	**+**	**+**	–	–	–	**+**	**+**	**+**	**+**	**+**	6
BJ-1F2	E	**+**	**+**	–	–	–	**–**	**–**	–	–	–	–	–	–	–	–	NA
BJ-1G9	prM	**+**	–	–	–	–	**–**	**–**	–	–	–	–	–	–	–	–	NA
BJ-2B1	prM, E	**+**	**+**	–	–	–	**–**	**–**	–	–	–	–	–	–	–	–	NA
BJ-2B11	E	**+**	**+**	–	–	–	**–**	**–**	–	–	–	–	–	–	–	–	NA
BJ-3A3	NS1	**+**	**+**	NA	NA	NA	NA	NA	–	–	–	–	–	–	–	–	NA
BJ-3E6	prM	**+**	**+**	–	–	–	–	–	–	–	–	–	–	–	–	–	NA
BJ-4A8	NS1	**+**	**+**	NA	NA	NA	NA	NA	–	–	–	–	–	–	–	–	NA
BJ-4C1	prM	**+**	–	–	–	–	–	–	–	–	–	–	–	–	–	–	NA
BJ-4D10	prM, E	**+**	**+**	–	–	–	–	–	–	–	–	–	–	–	–	–	NA
BJ-5B6	prM, E, prME	**+**	**+**	–	–	–	–	–	–	–	–	–	–	–	–	–	NA
BJ-5E5	NS1	**+**	**+**	NA	NA	NA	NA	NA	–	–	–	–	–	–	–	–	NA
BJ-5F7	NS1	**+**	**+**	NA	NA	NA	NA	NA	–	–	–	–	–	–	–	–	NA
BJ-5H4	E, prME	**+**	–	–	–	–	–	–	–	–	–	–	–	–	–	–	NA
BJ-6B11	E	**+**	**+**	–	–	–	–	–	–	–	–	–	–	–	–	–	NA
BJ-6E6	E	**+**	**+**	+	+	+	+	+	–	–	–	**+**	**+**	**+**	**+**	**+**	6
BJ-7C4	E, prME	**+**	**+**	–	–	–	–	–	–	–	–	–	–	–	–	–	NA
BJ-7G10	NS1	**+**	**+**	NA	NA	NA	NA	NA	–	–	–	–	–	–	–	–	NA
4G2†	VIF E	**–**	–	**+**	**+**	**+**	**+**	**–**	–	–	–	**+**	**+**	+	+	+	13
6B6C-1†	VIF E	**–**	–	**+**	**+**	**+**	**+**	**–**	–	–	ND	**+**	**+**	ND	ND	+	14
M2-1E7†	VIF E	**–**	–	**+**	**+**	**+**	**+**	**–**	–	–	ND	**+**	**+**	ND	ND	+	15
P3H8†	VIF E	**–**	–	**+**	**+**	**+**	**+**	**+**	–	–	ND	+	+	ND	ND	+	6
4A4	ZIKV E	**–**	–	–	–	–	**–**	**–**	–	–	–	–	–	–	–	+	6
17D7	WNV EDI	**–**	–	–	–	–	**–**	**–**	**–**	–	ND	–	**+**	ND	ND	–	61
4G4†	Flavivirus NS1	**+**	+	**+**	**+**	**+**	**+**	**+**	–	–	–	**+**	**+**	–	+	+	16
3.1112G	WNV NS1	**–**	–	–	–	–	**–**	**–**	–	–	–	–	**+**	–	–	–	62
10C6	MVE NS1	**–**	–	–	–	–	**–**	**–**	–	–	–	**+**	–	–	–	–	15
3G1*	dsRNA	+*	+*	ND	ND	ND	ND	ND	+	+	+	**+**	**+**	+*	+*	+*	49
2G4*	dsRNA	+*	+*	ND	ND	ND	ND	ND	+	+	+	**+**	**+**	+*	+*	+*	49
5G12^*d*^	PCV E	**–**	–	–	–	–	**–**	**–**	–	**+**	–	–	–	–	–	+	6
2G3.1	PaRV prM	**–**	ND	–	–	–	**–**	**–**	**+**	–	ND	–	–	ND	ND	ND	8
5B7	PaRV prM	**–**	ND	–	–	–	**–**	**–**	**+**	–	ND	–	–	ND	ND	ND	8
7D11	PaRV E	**–**	–	–	–	–	**–**	**–**	**+**	–	–	–	–	–	–	–	8
3G7	PaRV E	**–**	ND	–	–	–	**–**	**–**	**+**	–	ND	–	–	ND	ND	ND	8
2G10	PaRV E	**–**	ND	–	–	–	**–**	**–**	**+**	–	ND	–	–	ND	ND	ND	8
2D2/A3	PaRV E	**–**	ND	–	–	–	**–**	**–**	**+**	–	ND	–	–	ND	ND	ND	8
1E5	PaRV E	**–**	ND	–	–	–	**–**	**–**	**+**	–	ND	–	–	ND	ND	ND	8

a +, reactive; –, nonreactive; NA, not applicable; ND, not done. *, negative in acetone-fixed plates and positive in formaldehyde-fixed plates; †, pan-flavivirus reactive MAb.

b prME = 75-kDa protein; E = 50-kDa protein; prM = 25-kDa protein.

c The cutoff for a positive result was 2× OD of the same MAb dilution on mock-treated cells and >0.3. LAMV, ILOV, NHUV, CHAOV, and NOUV consisted of a chimeric virus containing the BinJV backbone and the prM and E genes from selected lineage II ISFs.

d Cross-reactive with ZIKV, as demonstrated previously (66).

To extend the lineage II ISF antigenic analysis, we constructed a series of chimeric viruses consisting of a BinJV genome backbone and the structural gene sequences (prM-E) of selected lineage II ISFs that represent both the *Aedes* and *Culex*-associated clades, including CHAOV, Lammi virus (LAMV), Ilomantsi virus (ILOV), Nhumirin virus (NHUV), and Nounané virus (NOUV) (17–21). We have previously shown that chimeric virus particles derived from the BinJV genome backbone with the prM-E genes substituted for those from a range of VIFs are structurally and antigenically indistinguishable from the wild-type VIF particle (6). This allowed us to assess the antigenic relationships between several lineage II viruses, even exotic viruses, for which we did not have access to isolates. The successful recovery of each chimeric virus was confirmed by reactivity with pan-flavivirus NS1-specific MAb 4G4 in immunofluorescence assays (IFA) and sequencing over the capsid-envelope and envelope-NS1 junctions (data not shown). Of the 16 BinJV-derived MAbs targeting E or prM, only the two cross-reactive MAbs (BJ-1E1 and BJ-6E6) recognized each of the BinJ/Lin II-prME chimeric viruses in a fixed-cell ELISA. When the panel of VIF-derived MAbs were also assessed with these chimeras, unlike BinJV and HVV, the prMEs of CHAOV, LAMV, ILOV, and NHUV were also recognized by the subset of cross-reactive MAbs that bind the E protein fusion peptide (4G2, 6B6C-1, M2-1E7, and P3H8), while the prME of NOUV was not detected by 4G2, 6B6C-1, or M2-1E7. Together, these results suggest that BinJV and HVV form a discrete antigenic type within the lineage II ISF group and are largely antigenically distinct from VIFs but that most lineage II ISFs share antigenic similarity with VIFs across the fusion peptide region.

### A single substitution in the BinJV fusion peptide ablates a conserved flavivirus-generic epitope in E domain II.

**FIG 2:**
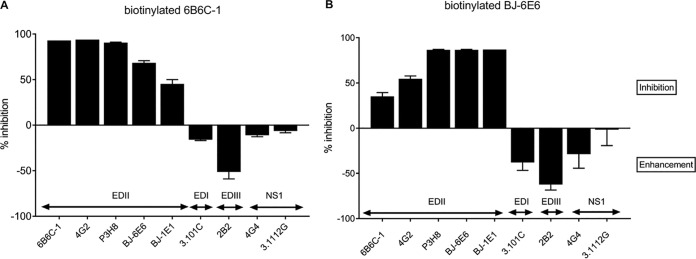
Competitive binding of cross-reactive BinJV MAbs BJ-6E6 and BJ-1E1. Competitive ELISA analysis of pan-flavivirus MAb 6B6C-1 (A) and BinJV cross-reactive MAb BJ-6E6 (B) was performed to determine whether the cross-reactive BinJV MAbs BJ-1E1 and BJ-6E6 were likely to bind the conserved epitope in the fusion peptide of domain II detected by other VIF cross-reactive MAbs.

**FIG 3:**
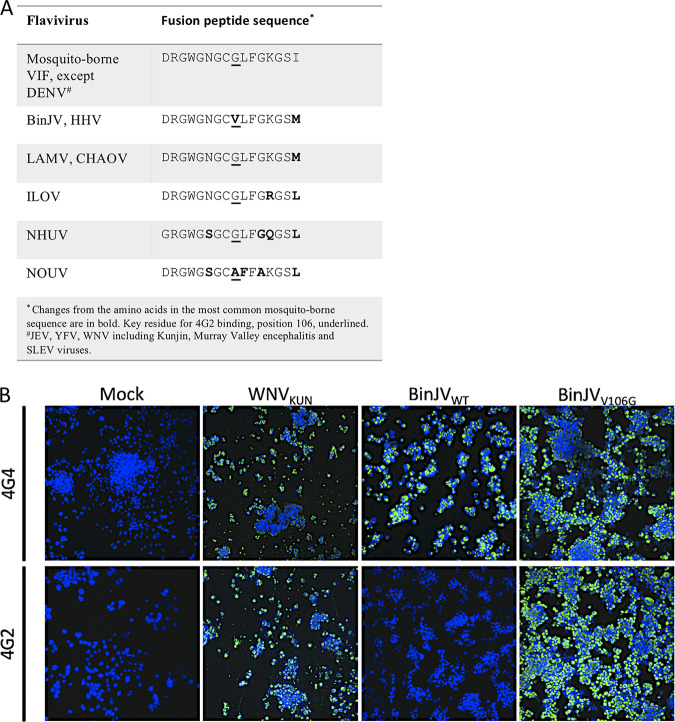
Lineage II ISF fusion peptide sequence analysis. (A) Homology of conserved flavivirus E domain II fusion peptide sequence of VIFs and selected lineage II ISFs. Variations from the VIF sequence are bolded, while key conserved residue for MAb 4G2 binding (E106) is underlined. (B) IFA staining of C6/36 infected with BinJV_V106G_, BinJV_WT_, and WNV_KUN_ (MOI of 1) or diluent (mock) and immunolabeled with anti-VIF E (4G2) or anti-VIF NS1 (4G4 and BinJV-reactive) MAb. Nuclei were stained with Hoechst 33342. Images were obtained at ×40 magnification.

The two BinJV-derived MAbs (BJ-1E1 and BJ-6E6) that are cross-reactive to VIFs were hypothesized to detect conserved epitopes in the same antigenic domain of the E protein detected by other VIF cross-reactive MAbs such as MAbs 4G2 and 6B6C-1 (22). This was confirmed when MAbs BJ-6E6 and BJ-1E1 partially inhibited biotinylated 6B6C-1 binding to its epitope in a competitive ELISA (and vice versa for 6B6C-1 and BJ-6E6), while strong inhibition of biotinylated BJ-6E6 binding by BJ-1E1 suggested that these MAbs have very similar contact residues (Fig. 2). However, the inability of MAbs 4G2, P3H8, and 6B6C-1 to bind BinJV and HVV was indicative of an amino acid substitution within the highly conserved fusion peptide in E domain II (DII) that has been defined as the major binding site for these antibodies (23). Indeed, sequence analysis revealed a substitution of a valine for a glycine at residue position 106 of the E protein in BinJV and HVV, compared to the consensus fusion peptide sequence for VIFs (Fig. 3A). This is consistent with previous studies that demonstrated a similar substitution at this residue in the WNV fusion peptide ablating reactivity of 4G2 and 6B6C-1 (23).

To confirm that a substitution in the fusion peptide of BinJV was responsible for the inability of MAbs 4G2 and 6B6C-1 to bind, infectious DNA of BinJV carrying a V106G substitution in E DII was generated by CPER (6, 8). When assessed in an IFA, MAb 4G2 was unreactive to wild-type BinJV but reacted strongly with BinJV_V106G_, while anti-NS1 MAb 4G4 reacted strongly to both viruses (Fig. 3B). These data confirm that the EDII G106V substitution in BinJV (and likely HVV) was responsible for the lack of binding of VIF-derived cross-reactive MAb 4G2.

### BinJV and HVV display a mosquito-restricted tropism.

While initial observations suggested that BinJV and HVV were insect specific, a wider range of vertebrate cells were assessed to confirm this (6). Our experiments showed that BinJV and HVV were unable to replicate in an extensive panel of vertebrate cell lines, including those derived from mammals, avians, amphibians, and reptiles, or cells of *Drosophila* origin after inoculation at a multiplicity (MOI) of 1 (Table 6 and Fig. S2). Furthermore, inoculation of embryonated chicken eggs with BinJV resulted in a decrease of at least 100-fold in infectious virus (compared to inoculum) in the chicken embryo homogenates 5 days after inoculation suggesting a lack of viral replication (Table S2). In contrast to Australian lineage I ISFs PCV, Parramatta River virus (PaRV), and Karumba virus (KRBV), BinJV and HVV infected and replicated efficiently in cells derived from a range of mosquito genera, including *Aedes-*, *Culex-*, and *Anopheles-*derived cell lines (Table 6 and Fig. S2) (24, 25). There was no consistent evidence of syncytium formation or cell death in C6/36 cells when inoculated with BinJV or HVV.

**TABLE 6:** Host range analysis of BinJV and HVV as determined by inoculation of several insect and vertebrate cell lines

Host organism	Cell line	Result^*a*^		
		BinJV	HVV	WNV_KUN_
Insect				
*Aedes albopictus*	C6/36	+	+	+
	RML-12	+	+	+
*Culex* spp.	Chao Ball	+	+	+
	HSU	+	ND	+
*Anopheles gambiae*	Mos.55	+	+	+
*Drosophila*	S2	–	–	+
				
Vertebrate				
Mammalian	BSR	–	–	+
	WT MEF	–	–	+
	IFNAR^–/–^ MEF	–	–	+
	Vero	–	–	+
	OK	–	ND	+
	SW-13	–	ND	+
Avian	DF-1	–	–	+
Amphibian	A6	–	ND	+
Reptilian	3CPL	–	ND	+
	VSW	–	ND	+

a +, positive by 4G4 staining in IFA; –, negative; ND, not done.

10.1128/mSphere.00095-20.2 FIG S2 Analysis of BinJV and HVV growth in insect and vertebrate cells. Insect cell lines (C6/36, RML-12, Chao Ball, Mos.55, and S2) and vertebrate cells (DF-1, WT MEFs, IFNAR ^−/−^ MEFs, OK, and SW-13) were inoculated with BinJV, HVV, or WNV at an MOI of 1 or mock infected and fixed at 5 days postinfection. IFA analysis was performed by probing with anti-flavivirus NS1 MAb 4G4. Nuclei were stained with Hoechst 33342. Images were obtained at ×20 magnification. Download FIG S2, TIF file, 2.5 MB. © Crown copyright 2020. 2020 Crown This content is distributed under the terms of the Creative Commons Attribution 4.0 International license.

10.1128/mSphere.00095-20.7 TABLE S2 Lack of replication of BinJV in embryonated chicken eggs. Download Table S2, DOCX file, 0.02 MB. © Crown copyright 2020. 2020 Crown This content is distributed under the terms of the Creative Commons Attribution 4.0 International license.

### BinJV replication is restricted after entry to vertebrate cells by innate immune responses in a temperature-dependent manner.

**FIG 4:**
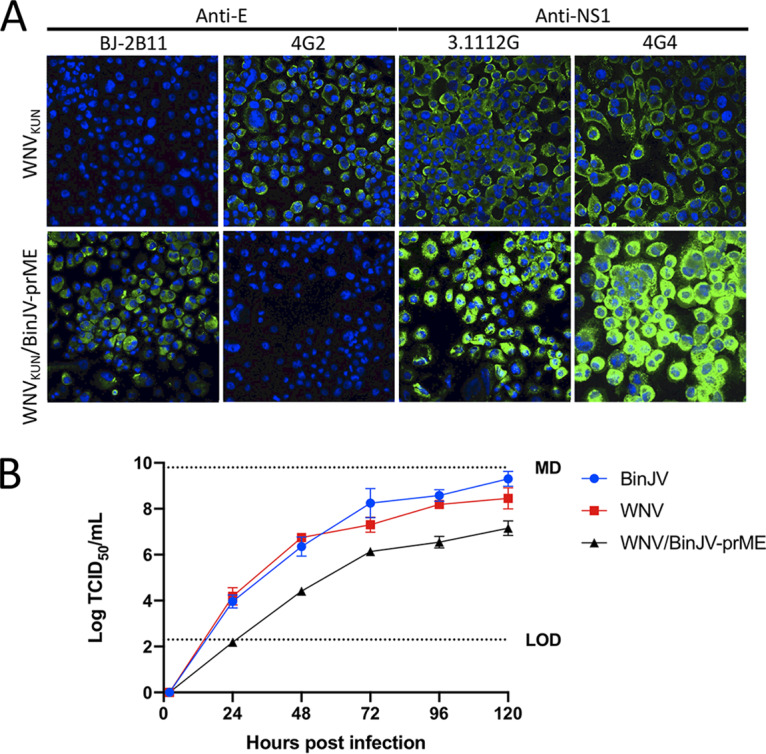
Generation of WNV_KUN_/BinJV-prME chimeric virus. (A) WNV_KUN_/BinJV-prME was generated using CPER. IFA was performed on fixed virus-infected C6/36 cells to confirm chimera recovery using anti-E MAbs BJ-2B11 (BinJV-specific) and 4G2 (VIF-reactive) and anti-NS1 MAbs 3.1112G (WNV-specific) and 4G4 (WNV and BinJV-reactive). Nuclei were stained with Hoechst 33342. Images were obtained at ×40 magnification. (B) Comparative growth kinetics of BinJV, WNV_KUN_, and WNV_KUN_/BinJV-prME in C6/36 cells infected at an MOI of 0.1. The levels of infectious virus were determined by TCID_50_ in C6/36 cells over 5 days.

While studies performed with the lineage I ISFs PCV and Niénokoué virus (NIEV) have revealed that replication of ISFs in vertebrate cells is restricted by pre- and post-cell entry blocks (7, 8), similar studies have yet to be performed on lineage II ISFs. To further investigate the stages in the vertebrate cellular replication cycle where lineage II ISFs replication is restricted, CPER was used to substitute the prM and E genes of WNV_KUN_ with those from BinJV to produce the chimeric virus WNV_KUN_/BinJV-prME. The recovery of viable chimeric virus was demonstrated by reactivity of MAbs specific to BinJV E (BJ-2B11) or to WNV NS1 (3.1112G) (Fig. 4A). Deep sequencing to confirm the identity of the chimeric virus identified two nucleotide changes at positions 3,102 (NS1) and 10,089 (NS5) which did not result in changes to the amino acid sequence. Replication kinetics demonstrated that the WNV_KUN_/BinJV-prME chimera replicated productively in C6/36 cells but grew less efficiently than the parental viruses, reaching titers 100- to 1,000-fold lower at each time point (Fig. 4B). The generation and characterization of the chimera of the opposite orientation (a BinJV infectious clone encoding the prM and E genes of WNV_KUN_ (BinJ/WNV_KUN_-prME), has been previously reported and shown to replicate with similar efficiency to WNV and BinJV in C6/36 cells (6). These chimeric viruses were then used in a series of experiments to detect inhibition of lineage II ISF replication at specific stages of vertebrate cell infection.

**FIG 5:**
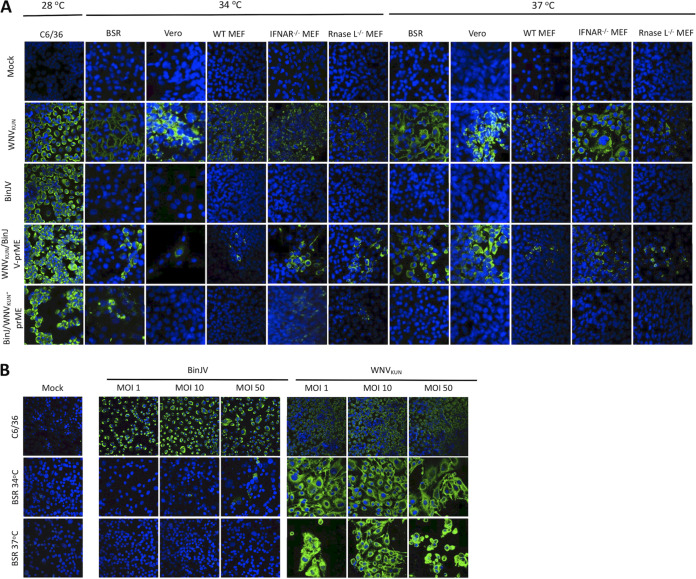
Host range restriction of BinJV in vertebrate cells. (A) Vertebrate cell lines were infected with BinJV, WNV_KUN_, BinJ/WNV_KUN_-prME, or WNV_KUN_/BinJV-prME or mock infected at an MOI of 1 and then incubated at 28°C (C6/36 cells) or 34°C or 37°C (vertebrate cells) before being fixed at 5 days postinfection. IFA analysis was performed by probing with the anti-flavivirus NS1 MAb 4G4. Nuclei were stained with Hoechst 33342. Images were obtained at ×40 magnification. (B) C6/36 and BSR cells were infected with BinJV or WNV_KUN_ at MOIs of 1, 10, and 50 and then incubated at 28°C (C6/36 cells) and 34°C or 37°C (BSR cells) and fixed at 5 days postinfection. IFA analysis was performed by probing with the anti-flavivirus NS1 MAb 4G4. Nuclei were stained with Hoechst 33342. Images were obtained at ×40 magnification.

To determine whether BinJV structural proteins in the chimeric virus could facilitate viral entry to vertebrate cells, a panel of mammalian cell lines (BSR cells, Vero cells, wild-type mouse embryonic fibroblasts [WT MEFs], interferon receptor knockout [IFNAR^−/−^] MEFs, and RNase L knockout [RNase L^−/−^] MEFs) were infected with WNV_KUN_/BinJV-prME and the parental viruses BinJV and WNV_KUN_ and then incubated at 34°C or 37°C. Both WNV_KUN_/BinJV-prME and WNV_KUN_ replicated in all vertebrate cell lines tested at both 34°C and 37°C, as determined by IFA staining. In comparison, wild-type BinJV failed to replicate in all inoculated vertebrate cell lines at either temperature (Fig. 5A). While these data indicate that BinJV structural genes are able to facilitate entry into vertebrate cells, it should be noted that fewer infected cells were observed qualitatively in several of the vertebrate cultures inoculated with WNV_KUN_/BinJV-prME compared to WNV_KUN_ (Fig. 5A). These data suggest that viral entry via the BinJV structural proteins may not be optimal in some vertebrate cells.

The ability of BinJV structural proteins to allow viral entry to a range of vertebrate cells indicated that a major restriction event for BinJV in vertebrate cells occurs after cellular entry. To investigate this, we inoculated the same cell lines with the chimeric virus derived from the BinJV genome backbone and containing the WNV_KUN_ structural (prM-E) proteins (BinJ/WNV_KUN_-prME) (6). Although no replication was observed in the majority of vertebrate cell lines assessed, limited replication of the BinJ/WNV_KUN_-prME chimera was seen in BSR cells when incubated at 34°C, although this was not consistent between replicates in multiple experiments (Fig. 5A). Inconsistent low-level replication of BinJ/WNV_KUN_-prME was also observed in MEFs lacking the receptors for type 1 interferon (IFNAR^−/−^ MEFs) or lacking RNase L activity (RNase L^−/−^ MEFs) but not in wild type MEFs incubated at that temperature (Fig. 5A). No replication of BinJ/WNV-prME was observed in any vertebrate cell line held at 37°C. If we assume that the WNV_KUN_ structural proteins on the surface of the chimeric virion provided efficient entry of the virus into vertebrate cells and release of genomic RNA into the cytoplasm, these data together suggest that host restriction of BinJV occurs postentry and is likely to be mediated by the innate immune response in a temperature-dependent manner. This is consistent with the trace levels of replication seen in some BSR cells at 34°C, since these cells also have a partial deficiency in the interferon response (26, 27). In contrast, BinJV replicated efficiently in C6/36 cells when incubated at 34°C (Fig. S5).

Due to the apparent suboptimal efficiency of the BinJV structural proteins to facilitate viral entry to some vertebrate cells (Fig. 5A), BSR cells were infected with wild-type BinJV at higher MOIs (1, 10, and 50) in an attempt to enhance viral entry to the cells and initiate BinJV replication. Although limited replication of BinJV was detected in BSR cells when infected at an MOI of 10 and incubated at 34°C (Fig. 5B), this was inconsistent between replicates of the experiment (Fig. S4). Indeed, only when the MOI was raised to 50 were trace levels of BinJV replication consistently detected in BSR cells, and only when incubated at 34°C (Fig. 5B). These data suggest that although the structural genes of BinJV (and presumably other lineage II ISFs) can facilitate entry into the vertebrate cell, this is relatively inefficient in comparison to VIFs. These data also confirm that replication of BinJV in vertebrate cells is temperature dependent and completely restricted at 37°C.

## DISCUSSION

While numerous lineage I ISFs have been reported in the Australian mosquito population (5, 9, 24, 25), the discovery of BinJV and the closely related HVV represent the first lineage II ISFs in Australia. ISFs are typically detected in a single mosquito genus and in most cases a single mosquito species (24), suggesting a high level of adaptation to their insect host. However, some exceptions do exist. CFAV, the prototype ISF, has been detected in both *Aedes* and *Culex* mosquitoes (28). Furthermore, lineage II ISFs CHAOV and Long Pine Key virus (LPKV) have been detected in *Aedes* (spp. *vexans*, *albopictus*, and *bekkui*), *Armigeres subalbatus*, and Culex pipiens mosquitoes or in *Anopheles crucians*, *Aedes atlanticus*, and Culex nigripalpus mosquitoes, respectively (20, 29–31). While BinJV and HVV were only detected in and isolated from *Aedes normanensis* and *Aedeomyia catasticta*, respectively, both viruses efficiently replicated in cell lines derived from *Aedes*, *Culex*, and *Anopheles* mosquitoes, suggesting a possible wider host range. Both BinJV and HVV failed to replicate in an extensive panel of vertebrate cell lines tested, grown at standard culturing temperature, as well as those derived from *Drosophila*, which is indicative of a virus that is mosquito specific. This extends the findings of similar reports on the host range of other lineage II ISFs (18, 21, 32).

The high prevalence in nature of some ISFs, such as PaRV, *Culex* flavivirus (CxFV), and KRBV (24, 25, 33), indicates that they are most likely transmitted vertically, although this also appears to be dependent on the season in which the mosquitoes are captured (34). In comparison, the prevalence of BinJV was relatively low. Only a single isolate was detected in more than 200 pools of *Aedes normanensis* mosquitoes that were collected at the same time and place, suggesting that the vertical transmission of BinJV may be relatively inefficient. *Aedes normanensis* mosquitoes are freshwater flood mosquitoes whose numbers rapidly increase in response to wet season rainfall (35). Thus, the sporadic appearance of this mosquito species from eggs hatched after flooding rains may require the virus to withstand prolonged periods of desiccation in infected eggs, reducing the infectious titer to trace levels below the sensitivity of detection in the assays used here. Other, more sensitive approaches may be needed to investigate this further. In addition, further laboratory studies to determine the mode and efficiency of transmission of BinJV between mosquitoes are also required.

In contrast to the low prevalence of BinJV, the data presented here suggest that HVV may be much more prevalent, with 60% (9/15) of the pools of *Aedeomyia* mosquitoes testing positive for this virus. Similar to *Aedes normanensis* mosquitoes, *Aedeomyia catasticta* mosquitoes are freshwater flood mosquitoes; however, their vector status for arbovirus transmission is considered to be insignificant (36). Although this particular mosquito species was commonly captured in the past, this species is no longer frequently captured, likely due to a switch from animal-baited traps to CO_2_-baited traps (37); thus, no recent data on the prevalence of this mosquito species are available. Screening of contemporary *Aedeomyia catasticta* collections to determine whether HVV has persisted in the population is warranted.

Prior to this report, there have been no comprehensive antigenic analyses of lineage II ISFs, and few reactive antibodies are available to use as research tools. For the first time, we have generated a large panel of MAbs to a lineage II ISF (BinJV) and analyzed their reactivity to other ISFs and VIFs. Because we did not have access to isolates of several exotic lineage II ISFs, including LAMV, NHUV, and ILOV, we used CPER to construct chimeric viruses by splicing the prM and E gene sequences of these viruses into the BinJV genome backbone. As we have previously shown by high-resolution cryo-EM (6), these chimeric virus particles authentically mimic the antigenic structure of the wild-type particle, allowing us to report this first investigation of the antigenic relationships between a large representative panel of lineage II ISFs with several other flaviviruses. The BinJV-, HVV-, and flavivirus-reactive MAbs produced here also provide a unique set of research tools for further studies on lineage II ISFs.

Despite sharing the insect-specific phenotype with their lineage I cousins, lineage II ISFs are genetically and antigenically more closely related to VIFs and are often recognized by flavivirus group-reactive MAbs directed to a highly conserved epitope in the fusion domain of the E protein that appears to be absent in lineage I ISFs (17, 19). Here, we demonstrate that a single amino acid substitution in the fusion domain of BinJV, also identified in a subset of other lineage II ISFs (HVV, NOUV, BJV, and LPKV), ablates the binding of group-reactive MAbs to this epitope. Mutating this residue back to a glycine reinstated binding of the group-reactive MAb 4G2 to BinJV, and presumably the same would occur with other lineage II ISFs that contain a similar substitution. The lack of this conserved residue in BinJV and HVV may explain why these viruses have not previously been detected by routine surveillance of mosquito-borne viruses in northern Australia, using MAb 4G2 to screen C6/36 cell cultures inoculated with mosquito samples (38).

Studies using chimeric viruses between PCV and WNV_KUN_, as well as between YFV and NIEV, have previously shown that lineage I ISF replication is blocked at both pre- and postentry stages in vertebrate cell infection (7, 8). Similar studies have not been reported for lineage II ISFs. In the present study, the use of chimeric viruses containing the replicative genes and UTRs of WNV_KUN_ and the prM and E genes of BinJV (WNV_KUN_/BinJV-prME) and vice versa (BinJ/WNV_KUN_-prME) allowed us to perform a similar investigation on BinJV. While the BinJV structural proteins could facilitate limited viral entry to vertebrate cells, the process was not efficient indicating that lineage II ISFs likely experience at least a partial block at this stage of infection. However, even when vertebrate cell entry was efficiently facilitated by the structural genes of WNV_KUN_, the BinJ/WNV_KUN_-prME chimera failed to replicate in vertebrate cells held at 37°C, even those that lacked components of the type 1 interferon response (IFNAR^−/−^ MEFs, RNase L^−/−^ MEFs, and Vero cells) (39, 40). This indicates that a major host restriction of BinJV occurs postentry and is likely mediated by interferon-independent mechanisms.

BinJV and BinJ/WNV_KUN_-prME failed to grow in reptile cell lines held between 28 and 34°C, while trace replication of BinJ/WNV_KUN_-prME was observed in a few mammal-derived cell lines (IFNAR^−/−^ MEFs, RNase L^−/−^ MEFs, and BSRs) held at 34°C. Consistent with its poor efficiency of cell entry, replication of BinJV could also be forced in BSRs under a very high MOI (i.e., MOI 50) but only at 34°C (not at 37°C). Together, these data suggest an additional temperature-dependent mechanism is at least partially involved in BinJV host restriction. We and others have previously observed a similar temperature restriction for other flaviviruses. Rabensburg virus (a divergent lineage of WNV) was shown only to replicate efficiently in vertebrate cells at or below 35°C (41, 42), whereas Bamaga virus (BgV; a new Australian flavivirus in the yellow fever virus group) was also restricted in most vertebrate cells incubated at temperatures above 36°C (43, 44). Further studies revealed that serial passage in vertebrate cells induced adaptive mutations in the nonstructural proteins of both viruses that facilitated efficient replication in vertebrate cells at 37°C (41, 44); however, the precise mechanisms involved are yet to be defined. In this context it should also be noted that previous studies with chikungunya virus have indicated that enhanced viral growth at lowered temperatures (35°C) are likely the result of a reduced interferon-mediated antiviral response (45). The ability of BinJV to replicate (albeit poorly) in some vertebrate cells lines held at lower temperatures provides novel insights into the vertebrate restriction factors of lineage II ISFs and extends the findings for the closely related ISFs LAMV and ILOV, which failed to replicate in human (HEK293), mouse (L929), monkey (Vero), toad (XTC), and snake (*Boa constrictor*) cells held at 37, 33, 30, and 27°C (18).

In conclusion, BinJV and HVV are the first lineage II ISFs found in Australia. Despite their closer genetic and antigenic relationship to VIFs, lineage II ISFs display a similar host restriction phenotype to lineage I ISFs. The creation of BinJV/WNV chimeras allowed us to establish that lineage II ISF host restriction likely occurs at multiple levels of the virus replication cycle in vertebrate cells, with blockages occurring pre- and postentry. While temperature-sensitive and IFN-independent mechanisms appear to play a major role in this host restriction, it is likely that these viruses have not evolved (or have lost) efficient mechanisms of resistance to vertebrate innate immune responses, and their replication is likely to be severely suppressed by vertebrate-specific antiviral factors. The suite of MAbs produced to BinJV in this study, some of which cross-react with other lineage II ISFs, provide a unique set of tools to further investigate this intriguing group of viruses, including their antigenic structure, mechanisms of host restriction, and modes of transmission.

## MATERIALS AND METHODS

### Cell culture.

C6/36 (Aedes albopictus, RNAi deficient) and Mos.55 (Anopheles gambiae) cell lines were maintained at 28°C in Roswell Park Memorial Institute 1640 (RPMI) medium and supplemented with 5% heat-inactivated fetal bovine serum (FBS). RML-12 (Aedes albopictus, RNAi competent), Chao Ball (*Culex tarsalis*), and HSU (Culex quinquefasciatus) cell lines were maintained in Leibovitz’s L-15 medium supplemented with 10% FBS and 10% tryptose phosphate broth. *Drosophila* S2 cells were maintained in Schneider’s *Drosophila* medium with 10% FBS.

Mammalian and avian cell lines used in this study were maintained at 37°C and 5% CO_2_, while reptilian cell lines were maintained at 28°C. BSR (*Mesocricetus auratus*, baby hamster kidney), Vero (Cercopithecus aethiops, African green monkey kidney), WT MEFs, IFNAR^−/−^ MEFs, and RNase L^−/−^ MEFs (Mus musculus, mouse embryonic fibroblast wild type, interferon knockout, and RNase L knockout), and OK (*Didelphis marsupialis virginiana*, opossum kidney, supplied by David Williams, CSIRO) were maintained in Dulbecco modified Eagle medium (DMEM) containing 5% FBS, while DF-1 (Gallus gallus, chicken embryo fibroblast, provided by David Williams, CSIRO) and SW-13 (Homo sapiens, human adrenal gland/cortex) were maintained in DMEM containing 10% FBS. VSW (*Daboia russelii*, viper epithelial, provided by David Williams, CSIRO) cells were maintained in minimum essential Eagle medium (MEM) with Hanks salts, 0.35 g/liter sodium bicarbonate, and 10% FBS. 3CPL (*Crocodylus porosus*, crocodile lung fibroblast, provided by Steven Davis) cells were maintained in medium 199 (M199), with 25 mM HEPES, Hanks salts, 10% FBS, and amphotericin B. A6 (Xenopus laevis, frog kidney epithelial) cells were maintained in 10% FBS RPMI. All media were supplemented with 50 U/ml penicillin, 50 μg/ml streptomycin, and 2 mmol/liter l-glutamine.

### Virus culture.

The following viruses were used here: WNV_KUN_ (GenBank AY274504), BinJV (GenBank MG587038), and HVV (GenBank MN954647). Virus stocks were generated by infecting vertebrate or C6/36 cell monolayers with virus at an MOI of 0.1 and incubation with rocking at 28°C for 1 h. After this, an inoculum was removed and replaced with fresh growth medium containing 2% FBS and incubated at 28°C for 5 dpi before harvesting by centrifuging at 3,000 rpm and 4°C for 10 min. Clarified supernatant was supplemented with additional FBS to increase the total concentration to 10%, aliquoted, and stored at –80°C until required. Virus stock titers were calculated based on the TCID_50_ (46), using methods previously described (47), after titration of stocks on C6/36 cells and staining by fixed-cell ELISA with the pan-flavivirus NS1 antibody 4G4 (16).

### Mosquito collection, detection, and isolation of BinJV and HVV.

The first detection of BinJV occurred from mosquitoes trapped in 2010 in Katherine, near the Binjari community, Northern Territory, Australia, in 2010. The trapping and processing of these mosquitoes, as well as viral RNA detection in mosquito homogenates, were detailed previously (9). Subsequent isolation of BinJV occurred from adult mosquitoes that were collected using CO_2_-baited light traps from the Bradshaw Field Training Area in the Northern Territory of Australia in 2014, as described previously (6, 48). In contrast, the first detection of HVV occurred through the deep sequencing of unidentified virus isolates. All other mosquito samples assessed for the presence of BinJV and HVV were archival or recent mosquito pools collected for various studies by the University of Western Australia or Northern Territory Department of Health, while colony-reared Aedes aegypti mosquitoes established from mosquitoes collected at Townsville and Cairns were provided by Major Weng Chow (Australian Defence Force Malaria and Infectious Disease Institute) and Jonathan Darbro (Queensland Institute for Medical Research–Berghofer), respectively.

Screening of mosquitoes for the presence of RNA viruses by mosquito homogenization, inoculation onto C6/36 cell monolayers, and subsequent MAVRIC (MAbs against viral RNA intermediates, see below) ELISA assessments were performed as described previously (25, 49). Specific details for the MAVRIC ELISA are provided below. MAVRIC-positive samples were assessed for the presence of BinJV or HVV by using reverse transcription-PCR and pan-flavivirus-specific primers (FU2 and cFD3) as described previously (10, 51). Pure BinJV and HVV stocks were generated by using the extracted RNA to transfect C6/36 cells, and the virus in the supernatant was expanded by passage in C6/36 cells with the supernatant harvested on day 7 postinoculation.

### MAVRIC ELISA.

The MAVRIC assay is a novel, high-throughput ELISA-based system that uses anti-double-stranded RNA MAbs to detect viral RNA replication complexes during the replication of positive-sense, single-stranded, and double-stranded RNA viruses. The system has been instrumental in detecting and isolating a number of novel Australian insect-specific viruses (5, 52–54). Fixed-cell ELISA was performed as previously detailed (16, 49), with incubation steps being performed at 37°C. Briefly, plates that had been fixed in a solution of 4% formaldehyde (vol/vol) and 0.5% Triton X-100 in PBS were blocked with ELISA blocking buffer (0.05 M Tris-HCl [pH 8.0], 1 mM EDTA, 0.15 M NaCl, 0.05% [vol/vol] Tween 20, 0.2% [wt/vol] casein) before probing with a cocktail of MAbs 3G1 and 2G4 (anti-dsRNA) diluted in blocking buffer. After incubation, the plates were washed with PBS containing 0.05% Tween 20 (PBST) prior to adding horseradish peroxidase (HRP)-conjugated goat anti-mouse Ig (P0447, Dako; diluted 1/2,000 to 1/3,000) and further incubated for 1 h before washing with PBST. Finally, substrate solution [1 mM 2,2-azino-bis(3-ethylbenzthiazoline-6-sulfonic acid) (ABTS) and 3 mM H_2_O_2_ in a buffer prepared by mixing 0.1 M citric acid with 0.2 M Na_2_HPO_4_ to give a pH of 4.2] was added to each well, and the plates were incubated in the dark at room temperature for 1 h. The absorbance was measured at 405 nm. Wells were considered positive if the optical densities were greater than twice the average of the mock-infected wells.

### Next-generation sequencing and phylogenetic analyses.

Sequencing of BinJV has previously been reported (6). The ORF of HVV was elucidated by extracting RNA from concentrated virions, using a Macherey-Nagel Nucleospin RNA virus RNA isolation kit (ref. 740956.250) per the manufacturer's instructions (Macherey-Nagel, Düren, Germany). Viral RNA was sequenced at the Australian Genome Research Facility (AGRF; Brisbane, Australia) using the Illumina HiSeq platform. The viral genome was assembled using Geneious R8 software.

Amino acid multiple sequence alignments over the ORFs of selected flavivirus sequences were performed with MAFFT v7.017, with the algorithm selected automatically, using a scoring matrix of BLOSUM62, a gap open penalty of 1.53, and an offset value of 0.123. FastTree 2.1.5 was then used to construct a tree which uses the maximum-likelihood approximation method, with an optimization for gamma20 likelihood selected. Branch support values were calculated using a Shimodaira-Hasegawa test. These analyses were done within the Geneious R8 package.

The genomes of BinJV and HVV were annotated according to guidelines from (4, 11, 12). For sequence analysis, pairwise nucleotide and amino acid identities between BinJV and HVV or multiple amino acid alignments between BinJV, HVV, and other flaviviruses were performed using the MUSCLE algorithm (55). Glycosylation sites were predicted with NetNGlyc (http://www.cbs.dtu.dk/services/NetNGlyc/), while transmembrane domains were predicted with TMHMM (http://www.cbs.dtu.dk/services/TMHMM/).

### Circular polymerase extension reaction.

Infectious DNA constructs of BinJV and between BinJV and WNV_KUN_ were generated according to previously described methods (8) (Fig. S3). Briefly, RNA was extracted from supernatant of infected C6/36 cells (BinJV) or from secreted virus particles of infectious clones (WNV_KUN_) (56, 57) and converted to cDNA using SuperScript IV reverse transcriptase (Thermo Fisher Scientific) according to the manufacturer’s instructions. The cDNA was then used as a template for a set of primer pairs to produce overlapping dsDNA fragments covering the BinJV genome or WNV_KUN_ backbone. To generate BinJ/ISF-prME chimeras, geneblocks for the exotic lineage II ISF prME (CHAOV, ILOV, LAMV, NHUV, and NOUV) region containing BinJV overhangs were designed (Table S3). A complete list of primers used can be found in Table S4 and S5). For each CPER assembly, 0.1 pmol of each viral cDNA fragment or geneblock was added to a Q5 PCR (NEB) according to the manufacturer’s instructions. The thermal cycling conditions were as follows: two cycles of 98°C for 30 s, 55°C for 30 s, and 72°C for 6 min; followed by 10 cycles of 98°C for 30 s, 55°C for 30 s, and 72°C for 6 min; and then holding at 14°C. The entire CPER reaction was transfected into C6/36 monolayers using Effectene (Qiagen) in accordance with the manufacturer’s instructions, and the passage 0 (P_0_) cell culture supernatants were harvested and stored at –80°C at 7 days posttransfection.

10.1128/mSphere.00095-20.3 FIG S3 Schematic of the CPER strategy to generate infection DNA of BinJV and chimeric viruses. BinJV, BinJ/Lin II ISF-prME, and WNV_KUN_/BinJV-prME chimeric viruses are generated by amplifying DNA fragments that share overlapping terminal regions before annealing together in CPER and transfecting mosquito cells with the reaction. Download FIG S3, TIF file, 2.7 MB. © Crown copyright 2020. 2020 Crown This content is distributed under the terms of the Creative Commons Attribution 4.0 International license.

10.1128/mSphere.00095-20.4 FIG S4 Host range restriction of BinJV in vertebrate cells. C6/36 and BSR cells were infected with BinJV or WNV_KUN_ at MOIs of 1, 10, and 50 and fixed at 5 days postinfection. IFA analysis was performed by probing with anti-flavivirus NS1 MAb 4G4. Nuclei were stained with Hoechst 33342. Images were obtained at ×40 magnification. Download FIG S4, TIF file, 0.7 MB. © Crown copyright 2020. 2020 Crown This content is distributed under the terms of the Creative Commons Attribution 4.0 International license.

10.1128/mSphere.00095-20.5 FIG S5 Temperature-dependent of BinJV and WNV_KUN_ in C6/36 cells. RNA derived from BinJV or WNV_KUN_ was transfected into C6/36 cells incubated at 28 or 34°C in triplicate before titration of the supernatants onto uninfected C6/36 cells and determination of the viral titers by TCID_50_. LOD, limit of detection. *P* > 0.05. Download FIG S5, TIF file, 1.3 MB. © Crown copyright 2020. 2020 Crown This content is distributed under the terms of the Creative Commons Attribution 4.0 International license.

10.1128/mSphere.00095-20.8 TABLE S3 Sequences for BinJ/Lin II ISF-prME GeneBlocksLin II ISF-prME GeneBlocks. Download Table S3, DOCX file, 0.02 MB. © Crown copyright 2020. 2020 Crown This content is distributed under the terms of the Creative Commons Attribution 4.0 International license.

10.1128/mSphere.00095-20.9 TABLE S4 Fragments and primer sets used to generate BinJV CPER constructs. Download Table S4, DOCX file, 0.02 MB. © Crown copyright 2020. 2020 Crown This content is distributed under the terms of the Creative Commons Attribution 4.0 International license.

10.1128/mSphere.00095-20.10 TABLE S5 Primer sets used to generate chimeric virus constructs. Download Table S5, DOCX file, 0.02 MB. © Crown copyright 2020. 2020 Crown This content is distributed under the terms of the Creative Commons Attribution 4.0 International license.

To confirm the presence of replicating virus after CPER, preliminary analysis was confirmed by trypsin treating the P_0_ C6/36 monolayers and seeding the cells onto glass coverslips, followed by incubation for a further 3 days and then fixing the monolayers in 100% ice-cold acetone. IFA analysis was performed using virus-specific E and NS1 MAbs. To confirm virus identities, deep sequencing of the WNV_KUN_/BinJV-prME chimera was performed on RNA extracted from concentrated virions as described above, while BinJ/ISF-prME chimeras were Sanger sequenced over the C/prM and E/NS1 junctions (AGRF).

### MAb production.

To generate MAbs that were specific to BinJV, BALB/c mice (Animal Resources Centre, Murdoch, Western Australia) were immunized twice via the subcutaneous route with purified BinJV virions, along with the inulin-based adjuvant Advax (Vaxine, Ltd., Adelaide, Australia). The mice were boosted with purified BinJV virions by intravenous injection 4 days prior to harvesting of the spleen. All animal procedures were approved by the University of Queensland Animal Ethics Committee and conducted in accordance with the *Australian Code of Practice for the Care and Use of Animals for Scientific Purposes.* Fusion of the spleen cells with NS0 myeloma cells (European Collection of Cell Cultures) was performed as previously described (9, 50). Hybridomas secreting BinJV-reactive antibodies were identified by fixed-cell ELISA as previously described (16). BinJV-reactive antibodies were then further analyzed for reactivity using Western blots and/or fixed-cell ELISAs for cross-reactivity to other flaviviruses (PCV, PaRV, CFAV, MVEV, BgV, WNV, DENV-2, and ZIKV) with incubation steps being performed at 37°C using methods previously described. The neutralization capabilities of each MAb was assessed using methods previously detailed (58, 59). The isotype of each MAb was determined using mouse MAb isotyping reagents (Sigma-Aldrich, St. Louis, MO) according to the manufacturer’s instructions.

### Antigenic assessment and cross-reactivity analyses.

To assess the antigenic similarities of flaviviruses involved in this study to VIFs and lineage I ISFs, C6/36 cell monolayers grown in 96-well plates were infected with selected VIFs representing each of the mosquito-borne flavivirus serogroups (WNV_KUN_, MVEV, ZIKV, DENV-2, and BgV) and Australian ISFs (PCV, PaRV, and CFAV). The reactivity of an extensive panel of MAbs to each of the viruses was then assessed by a fixed-cell ELISA as detailed above.

### Competitive ELISA.

ELISA plates (Greiner high binding) were coated with 100 ng/well of purified MAb 3.67G (62) and incubated at 4°C overnight in PBS. The MAbs were purified using protein A columns (GE Life Sciences) according to the manufacturer’s instructions. After washing with PBST, the plates were blocked with ELISA blocking buffer and WNV_KUN_ virions in crude C6/36 culture supernatant was added and incubated for 1 h at 37°C. After washing with PBS/T, a saturating concentration of unlabeled MAbs as hybridoma culture fluid was added in triplicate wells, followed by incubation at 37°C. Without removing the unlabeled MAbs, a predetermined nonsaturating concentration of biotinylated MAb 6B6C-1 or BJ-6E6 (prepared using a B-tag biotinylation kit [Sigma-Aldrich] according to the manufacturer’s instructions) was added, and the plates incubated for a further 1 h at 37°C. After a washing step with PBS/T, bound biotinylated MAb was detected with HRP-conjugated streptavidin and incubation at 37°C for 30 min. After a final wash, ABTS substrate solution was added as above.

### *In vitro* host range assessment.

To assess the *in vitro* host range of BinJV and HVV, vertebrate and insect cell infection assays were performed as previously detailed (9, 25, 52, 53). Briefly, monolayers of C6/36 cells and selected vertebrate cells were grown on glass coverslips before inoculating with virus at an MOI of 1. After incubation for 1 h with rocking, the inoculum was removed, and the coverslips were washed three times with sterile PBS before adding appropriate growth media back onto the coverslips. The coverslips were cultured for 5 days postinfection; the supernatant was then harvested and stored at –80°C, and the coverslips were fixed in 100% ice-cold acetone.

IFA analysis to assess virus replication was then performed as previously detailed (49), with all steps performed at 28°C with rocking. After blocking for 1 h, the coverslips were probed with flavivirus NS1 MAb 4G4 for a further 1 h before three washes with PBST. Coverslips were then probed with a 1/500 dilution of Alexa Fluor 488-conjugated goat anti-mouse IgG (Thermo Fisher Scientific) for 1 h before removal of the inoculum and incubation with a 1/1,000 dilution of Hoechst 33342 (Thermo Fisher Scientific) for a further 5 min. Coverslips were then washed three more times with PBST before being mounted on microscope slides with ProLong Gold (Thermo Fisher Scientific) and viewed under a LSM510 confocal microscope.

### Infections in embryonated chicken eggs.

The chicken embryos were obtained commercially from the Darwalla Group, Queensland, Australia. The age of embryonation was 10 days. The embryos were culled by freezing for 45 to 60 min at –20°C.

The growth of the virus in embryonated chicken eggs was assessed using a modification of a previously published method (60). Briefly, 50 μl of diluted virus were inoculated intravenously into three groups of three 9- to 12-day-old embryonated chicken eggs at the following doses: 10^7^, 10^6^, and 10^5^ TCID_50_ IU/ml (i.e., 10^5.7^, 10^4.7^, and 10^3.7^ TCID_50_ IU/egg, respectively). The eggs were incubated at 33 to 35°C and candled daily. Embryos that died between days 2 and 5 were retained at 4°C, and embryos remaining alive at 5 days postinfection were culled. Whole embryos were homogenized after removal of their heads in approximately 10 ml of heart-brain broth, after pooling the embryos inoculated with the same dose and culled at 5 days postinfection. The debris was removed by centrifugation, and the resulting approximate 5 ml of clarified egg homogenates was titrated by TCID_50_ on C6/36 cells as described above, incubated 5 days at 28°C, and analyzed by fixed-cell ELISA.

### Viral growth kinetics.

Viral growth kinetics in C6/36 cells was performed as described previously (8, 59). Briefly, confluent cell monolayers were inoculated in triplicate with wild-type parental virus (BinJV_WT_ and WNV_KUN_) or CPER-derived virus (WNV_KUN_/BinJV-prME) at an MOI of 0.1. After incubation at 28°C for 1 h, the inoculum was removed, and the monolayer was washed three times with sterile PBS before being replenished with growth media (2% FBS and RPMI). The supernatant was harvested at 2, 24, 48, 72, 96, and 120 h postinfection and stored at –80°C. Viral titers for each time point were determined based on a TCID_50_ assay of C6/36 cells, as described above, except that four wells were used for each dilution (25, 46, 59), before a two-way analysis of variance using GraphPad Prism was performed.

### Temperature dependence studies.

Monolayers of C6/36, BSR , Vero, and MEF (WT, IFNAR^–/–^, and RnaseL^–/–^) cells grown on glass coverslips were infected with BinJV, WNV_KUN_, WNV_KUN_/BinJV-prME, and BinJ/WNV_KUN_-prME at an MOI of 1, or mock infected, while BSR cells were also infected with BinJV and WNV_KUN_ at MOIs of 10 and 50 as detailed above. Incubation was performed at 28°C (C6/36 cells) or 34°C and 37°C (vertebrate cells), prior to fixing at 5 days postinfection and staining as detailed above.

### Data availability.

The GenBank accession numbers for the Binjari and Hidden Valley virus coding sequences are MG587038 and MN954647, respectively.
