# Supplementary material for: Antigenic Characterization of New Lineage II Insect-Specific Flaviviruses in Australian Mosquitoes and Identification of Host Restriction Factors
Source: mSphere. 2020 Jun 17;5(3):e00095-20. doi: 10.1128/mSphere.00095-20 (PMC7300350; doi:10.1128/mSphere.00095-20)
Supplement: TABLE S1 [file mSphere.00095-20-st001.docx]

| Table S1 Summary of mosquito pools screened for presence of BinJV and HVV | | | |
| --- | --- | --- | --- |
| Mosquito species | **Year of Collection** | **Number of Pools Screened^*^** | **BinJV/HVV isolations^#§^** |
| Peel Region, Western Australia | | | |
| *Aedes camptorhynchus* | 1988 | 10 | 0 |
| *Aedes alboannulatus* | 1988 | 1 | 0 |
| *Culex species* | 1988 | 1 | 0 |
| *Aedes camptorhynchus* | 1990 | 22 | 0 |
| *Aedes alboannulatus* | 1990 | 4 | 0 |
| *Aedes clelandi* | 1990 | 7 | 0 |
| *Aedes notoscriptus* | 1990 | 1 | 0 |
| *Aedes ratcliffei* | 1990 | 5 | 0 |
| *Aedes camptorhynchus* | 1991 | 2 | 0 |
| Leschenault, Western Australia | | | |
| *Aedes camptorhynchus* | 1988 | 6 | 0 |
| *Aedes alboannulatus* | 1988 | 1 | 0 |
| *Culex species* | 1988 | 2 | 0 |
| *Aedes camptorhynchus* | 1990 | 22 | 0 |
| *Aedes clelandi* | 1990 | 4 | 0 |
| *Aedes ratcliffei* | 1990 | 2 | 0 |
| *Aedes hesperonotius* | 1990 | 1 | 0 |
| *Aedes alboannulatus* | 1990 | 1 | 0 |
| *Culex species* | 1990 | 1 | 0 |
| *Aedes camptorhynchus* | 1991 | 1 | 0 |
| Cockburn, Western Australia | | | |
| *Coquillettidia species* | 1990 | 1 | 0 |
| Perth Metro, Western Australia | | | |
| *Aedes notoscriptus* | 1990 | 1 | 0 |
| Kununurra, Western Australia | | | |
| *Aedeomyia catasticta* | **1973** | **1** | **2 (HVV)** |
| *Aedeomyia catasticta* | **1974** | **2** | **1 (HVV)** |
| *Aedeomyia catasticta* | **1975** | **7** | **5 (HVV)** |
| *Aedeomyia catasticta* | **1976** | **5** | **2 (HVV)** |
| *Aedes normanensis* | 2014 | 35 | 0 |
| Wyndham, Western Australia | | | |
| *Aedes normanensis* | 2014 | 6 | 0 |
| Parry’s creek, Western Australia | | | |
| *Aedes normanensis* | 2014 | 10 | 0 |
| Pilbara region, Western Australia | | | |
| *Aedes normanensis* | 2014 | 12 | 0 |
| Darwin, Northern Territory | | | |
| *Aedeomyia catastica* | 2010 | 1 | 0 |
| *Aedes alboscutellatus* | 2010 | 1 | 0 |
| *Aedes eidsuoldensis* | 2010 | 1 | 0 |
| *Aedes kochi* | 2010 | 4 | 0 |
| *Aedes normanensis* | 2010 | 57 | 0 |
| *Aedes vigilax* | 2010 | 2 | 0 |
| *Anopheles bancroftii* | 2010 | 8 | 0 |
| *Anopheles farauti* | 2010 | 4 | 0 |
| *Culex quinquefasciatus* | 2010 | 1 | 0 |
| *Coquillettidia xanthogaster* | 2010 | 17 | 0 |
| *Culex annulirostris* | 2010 | 15 | 0 |
| *Culex gelidus* | 2010 | 1 | 0 |
| *Culex palpalis* | 2010 | 1 | 0 |
| *Culex vishnui* | 2010 | 1 | 0 |
| *Mansonia uniformis* | 2010 | 1 | 0 |
| Jabiru, Northern Territory | | | |
| *Coquillettidia xanthogaster* | 2010 | 1 | 0 |
| *Culex annulirostris* | 2010 | 1 | 0 |
| Bradshaw Field Training Area, Northern Territory | | | |
| *Aedes normanensis* | 2013 | 232 | **1 (BinJV)** |
| *Aedes vigilax* | 2013 | 5 | 0 |
| *Culex species* | 2013 | 9 | 0 |
| *Anopheles species* | 2013 | 36 | 0 |
| Katherine, Northern Territory | | | |
| *Aedes normanensis* | 2010 | 3 | **1 (BinJV)^a^** |
| Alice Springs, Northern Territory | | | |
| *Culex annulirostris* | 2010 | 21 | 0 |
| *Culex Spp* | 2010 | 1 | 0 |
| McArthur River Mine, Northern Territory | | | |
| *Aedes normanensis* | 2010 | 2 | 0 |
| *Aedes vigilax* | 2010 | 1 | 0 |
| *Culex annulirostris* | 2010 | 1 | 0 |
| *Culex sitiens* | 2010 | 1 | 0 |
| Alyangula Groote Eylandt | | | |
| *Aedes vigilax* | 2010 | 1 | 0 |
| Cairns, Queensland^b^ | | | |
| *Aedes aegypti* | 2015/2016 | 4 | 0 |
| Townsville, Queensland^b^ | | | |
| *Aedes aegypti* | 2016 | 12 | 0 |
| ^*^Pools sizes ranged between 1-107 mosquitoes  ^#^Assessed via MAVRIC, cut-ff for a positive result was 2xod of the same dilution of mAb on mock cells  ^§C^onfirmed by rt-pcr  ^a^BinJV RNA detected only – no isolate  ^b^ Colony-reared *Aedes aegypti* established from mosquitoes captured in Cairns and Townsville | | | |
