# Supplementary figures and images for: Antigenic Characterization of New Lineage II Insect-Specific Flaviviruses in Australian Mosquitoes and Identification of Host Restriction Factors
Source: mSphere. 2020 Jun 17;5(3):e00095-20. doi: 10.1128/mSphere.00095-20 (PMC7300350; doi:10.1128/mSphere.00095-20)

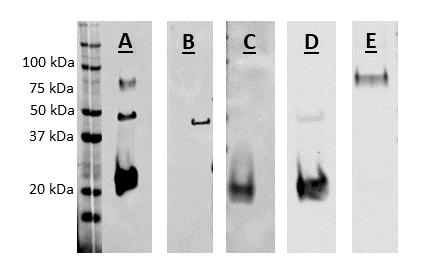

Supplement: FIG S1 [file mSphere.00095-20-sf001.tif]

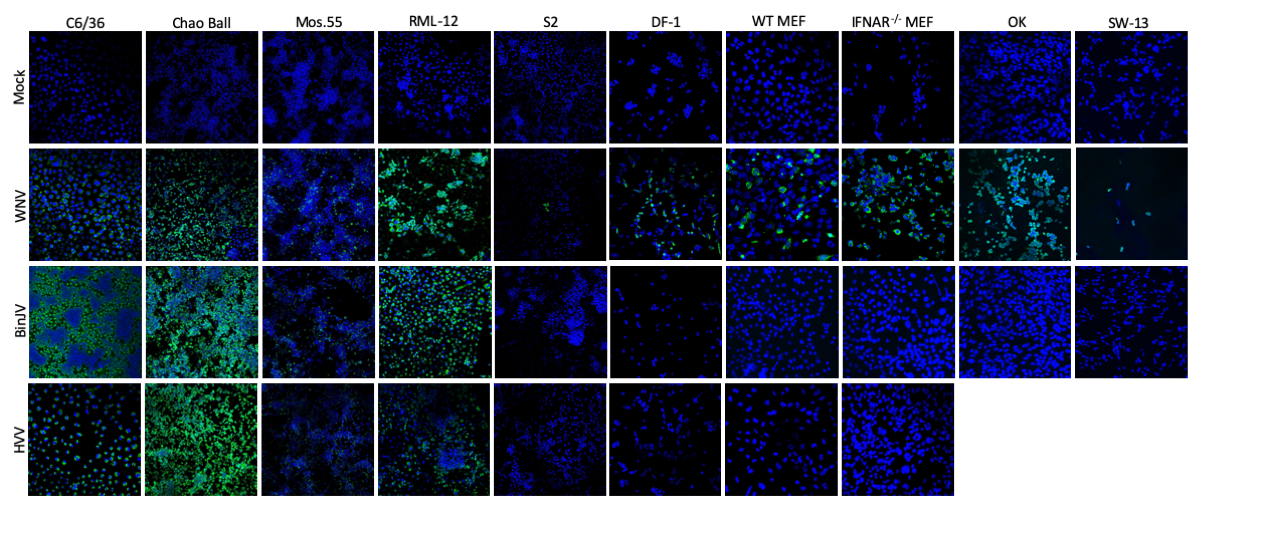

Supplement: FIG S2 [file mSphere.00095-20-sf002.tif]

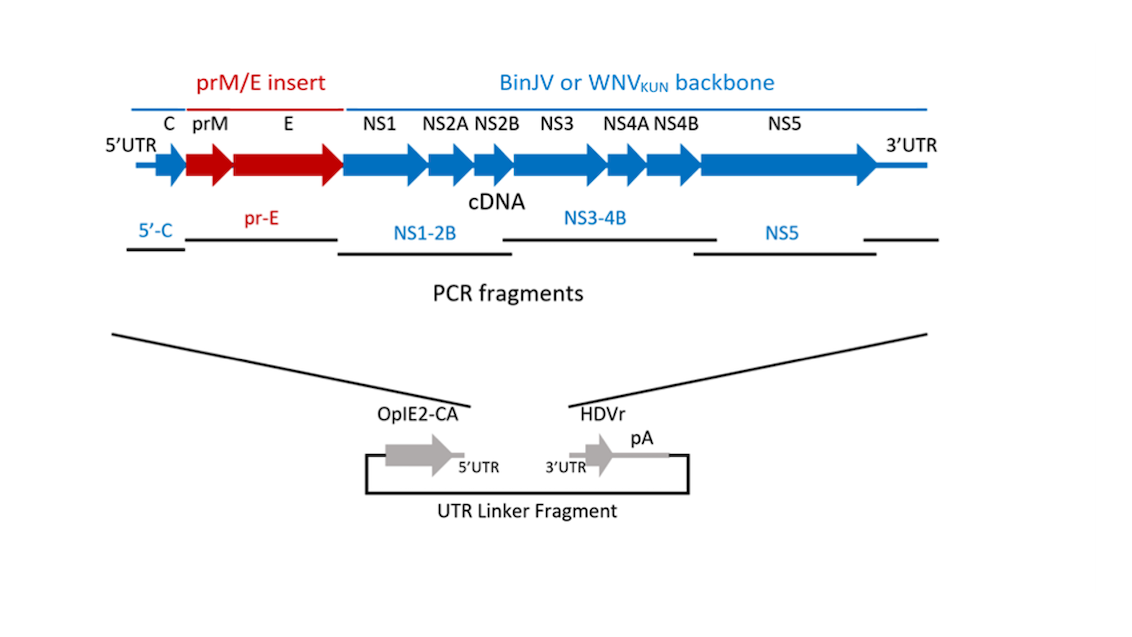

Supplement: FIG S3 [file mSphere.00095-20-sf003.tif]

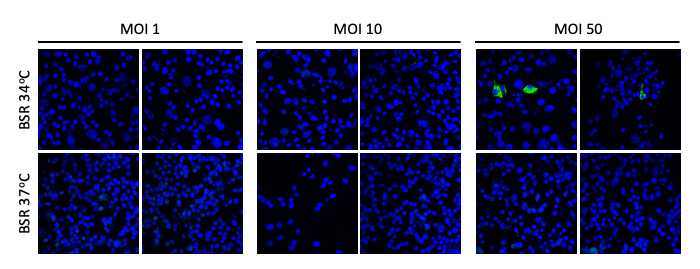

Supplement: FIG S4 [file mSphere.00095-20-sf004.tif]

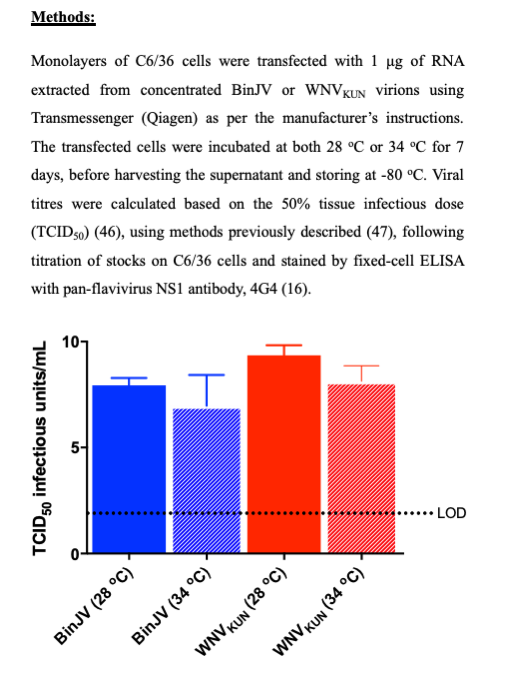

Supplement: FIG S5 [file mSphere.00095-20-sf005.tif]
