# Supplementary material for: Antigenic Characterization of New Lineage II Insect-Specific Flaviviruses in Australian Mosquitoes and Identification of Host Restriction Factors
Source: mSphere. 2020 Jun 17;5(3):e00095-20. doi: 10.1128/mSphere.00095-20 (PMC7300350; doi:10.1128/mSphere.00095-20)
Supplement: TABLE S2 [file mSphere.00095-20-st002.docx]

| Table S2: Lack of replication on BinJV in embryonated chicken eggs | | |
| --- | --- | --- |
| Total BinJV infectious units in inoculum for 3 chicken embryos (150ul) | **Total BinJV infectious units in pooled homogenates of 3 chicken embryos (5ml), 5 d.p.i** | **Interpretation** |
| 10^6.3^ | 10^3.7^ | Negative* |
| 10^5.3^ | 10^3.2^ | Negative |
| 10^4.3^ | <10^1.7^ | Negative |
| * The 100-400 fold decrease in total infectious virus in egg homogenates compared to the inoculum was indicative of no replication | | |
