# Supplementary material for: Antigenic Characterization of New Lineage II Insect-Specific Flaviviruses in Australian Mosquitoes and Identification of Host Restriction Factors
Source: mSphere. 2020 Jun 17;5(3):e00095-20. doi: 10.1128/mSphere.00095-20 (PMC7300350; doi:10.1128/mSphere.00095-20)
Supplement: TABLE S3 [file mSphere.00095-20-st003.docx]

**Table S3:** Sequences for BinJ/Lin II ISF-prME GeneBlocks

| **Chimera** | **Geneblock Sequence*** |
| --- | --- |
| BinJ/CHAOV-prME | GCTGCTCGTTGGAGCAGGAGCGATGGCTGCCACCAGGTTCACCAGAAATGGGTTTGTGTACATGAACGTCACTGGGAGTGATGTTGGGACCTGGTTATCCATTAAGACAGCAGTCGGGAACGGATCATGCATCGTTATGGCTACAGATGTGGGGACTTGGTGTGAAGACACTGTTACGTATCTTTGTCCCAAATTGGACGGTGCTGCTGAGCCTGACGACATTGATTGTTGGTGTAAAGTTGTATCAGTTTATGTTACCTATGGTAGGTGTAGACGAGATGGGGTGAGTAGACGCAGCAGACGATCTGTTGCTCTCGCACCGCATGGGACAGGAAACCTTCACACAGGAGAGGCACCAATGTGGAAATCACACACTGACGCTAGTAAATACTTGCAGCGAGTCGAACGGTGGGCTCTGAGAAATCCAGGATATCTTGGAATATTAGTGGCTATCGGATGGCTATTGGGCAGGACTACAGCGCAGCGTGTCATCTACATCACCCTCCTTGTGTTGATCGGACCAGCATACAGTCTGCAATGCATAGATGTGGCTAAGCGCGATTTTATCCAGGGAGTCAGTGGTGGCACATGGGTGGACGTCGTTTTGGACGTGCACGGGTGTGTGACTATTGCAGCAGAAGGTAAACCAACAGTGGATTTCAAGTTGACGAAGTTGGAGATGACGAAACTTGCAAAGGTGCGAGCGTACTGCCTCACTGCCTCCGTATCAGACATCACAGTCGAAAGTGGATGTCCAGGAACAGGCGAAATTCATAACACCAAGGCAAAGGACACCTCCTACATGTGCAAGGTGAGCTACCCTGACAGAGGTTGGGGAAATGGATGTGGACTGTTTGGAAAAGGGTCAATGGAAACGTGTGCTAAATTTGCTTGCACCAAGCAACTGCATGGACATGTAATTAGCCGCGAGAACATCGAAGCGGAAGTGGATATTTCTATCCATGGACAATCGGCTCCTGATTCTGACGATGCATCCAAGCGCAAGAACCGGAAGGAATTAGCCACGGCCACCATCACTCCTCAAGCTAGCTCAATAGAGGCGGATATGGGTGATTTTGGTAAAGTGGGTATGGACTGTTCTCTGGACATTGGAATTGACTTTGAGCAAGTCTTGATCGTAGACACAGCCTCACGCTGGTGGATGGTCAAGAGAGACTGGTTCCAGGACTTAGCACTTCCATGGACATCACCGTCAGCAGACTTCTGGCATGATCGTGACCGGTTAGTTGAATTTGGAGTTCCGCATGCTACGCGACAATCTGTATACTCCATTGGAGATCAAGAAGGAGCATTTTTCACTGCCGTGGCAAAAGCCCCGTCAGTCGAGTGGAACTCTGACAAAGTCAAGTTAGCGACCGGATTCCTGAAATGCAGAATCAAGCTTGGCAACATGAAGCTGAAAGGGTCAACATACGTCACGTGTGCGCAAGCGTTCACCTTTGCGAAGCGGCCAGTGGACACAGGTCATGGGACAGTCGTGTTCCAAGTATCATATGCTGGAACAGATGCGCCATGTAAAATACCAGTGGCCGTGACTGACAAACCAAACGGCGAGCATGTCGGGAGATTGGTGACAGCACACCCATTCATAGCGAAACAGAATGAGAAGGCAGTCGTTGAAGTTGAACCACCATTTGGAGACAGCTATATAGAGATTGGAGCAGGAACCACAAAAATCTCTGAAGCCTGGCACAAACCCGGAAGCTCCATTGGTAATGCGTTGGCCTTGTCATACAAAGGAATGAAACGGATAACAGCAATGGGCGAACACGCATGGGACTTTGGGTCCATCGGAGGATTCTTCAGCAGCATGGGAAAAGCGGTACATCACGTTTTTGGCAGTTTGTTCAGGACTCTATTTGGAGGCATTGGATGGATGGCAAAAATTTTGATTGGAGCGCTGCTCATTTGGCTAGGAATAAGCACCAGAGATCGCATGCTTGCCACATCTTTCATTCTGACTGGATCAATCCTGCTCTATTTGGCAACCACAACAGTTGGAGTGTCTGAAATAGGATGCAGTCTGGACATTAGCCGAAAAG |
| BinJ/ILOV-prME | GCTGCTGTTGGAGCAGGAGCGATGGCTACAACGGTGACCACCAGGGATGGAACGGTGTATGTGACCATGGCCCCGCAAGATGTCGGAAAATGGCTGGCGATAAGATCTAGACTTGGCAACTCGTCGTGTATATTGAACGCCATGGACGTTGGAAGCATGTGCGATGACAGCATCACTTATGAGTGTCCCGTCATTAATGACGGTACAGATCCTGAGGATATTGACTGTTACTGCAAAGGCCTCCCAATAGTCGTCACATATGGACGTTGTAAAAACGCGACAGGCGCAACCACGAAACCAACCAATCGGCGTTCACGCCGGTCAATAGCTCTAGCCCCACATGGGACCGGAGGTTTGCATCATGGAGACGCAGTCACATATAAAACGAACAATGCCAAGCGATTCTTGATGCGTCTGGAAAATTGGGCGCTGCGGAATCCTGGATATGTAGCTGTTATTCTCATATTGAGTTGGATGATGGGAAACACAAACAAGCAGAGAGCAGTGTACGTGCTGCTCATGCTGATGATTGCCCCGGTGTATGGACACCATTGCTCTGGAGTTAGCAAACGAGATTTCATTCAAGGAGTTAGTGGAGGAACATGGGTAGATCTGGTGTTGGACACACAAACATGCGTCACAATTGTGACGCCAGGCAAGCCTACGTTTGATTTCAAGTTGAACAAAATCGAGATCTCCAAACTGGCCAAGGTCCGCGAATACTGTCTTCAAGCTTCTATAACGGACACCACAACTGTAGCTGGATGTCCTAGCACGACTGAAGCGCATAACGACAAACGGAAAGATGCGTTGTATCTGTGCGAACGGAGCTATCCAGACAGAGGATGGGGAAATGGATGTGGTCTATTTGGAAGAGGAAGTTTGGACACATGCGCAAAATTTGCTTGTTCAAAGAAATTTTCAGGACATATGCTACAACGTGAAAACCTTGCAGTCGTCATCACGATGGCAGTGCAGGGAGGATCAGGAGCCACTGGTGATGATTCAACCAAGCGTAAATCACGCAATGAACTAGCTGAAGTCACTGTGACCCCTCAAGCGCCATATGTGGAAGGAGATTTTGCTGACTATGGCAAGGTTGGGTTGGAATGCTCAATTGACGTAGGAGTTGACATCAATGAGGTGTATACAGGTGATGCCGGTGGAAAATGGTGGATGGTCAAGCGAGCCTGGTTTCAGGATTTGGCTCTCCCATGGTCATCCCCCGCGGCAGACTTCTGGCATGATCGTGATCGACTAATGGAATGGGGAACCCCTCATGCAACTAAACAAAGTGTATACACACTTGGAGACCAAGAGGGTTCACTTATATCTTCCCTGGCCGACGCTCCCTCAATTGTGTTCAATACAGACAAAGTGGAATTTGAAGTGGGAAGAGTTAAATGCCGTGTTAAAATGGAAAATGCAAAATTGAAAGGTTCGACATATCTCATGTGCAAGCAAGCTTTCACATTTGAGAAGAGGCCTGTGGCTACCAACCATGGAACCGTCATCTTTCAAGTGAAATATGCCAACGCCGATGCGCCTTGCCGTGTGCCAGTTGCCATAAAAGAACTGCACGGTGCTCCAATAGTAGGAGGGCTTGTCTCATACCATCCTATAGTCTTGAAACAAAACGATGTTGTCACCATAGAAATTGAACCTCCTTTTGGAGACAGTGTCATTGAAGTAGGTGATGATGCGGCTAAGCTAACAGAAGCCTGGCACCGGGAGGGTAGTTCCATTGGAGAGGCTTTCCATAAGACTATGAAAGGAATACAACGGCTGACCGTCATGGGTGATGCCGCCTGGGACTTTGGTTCTGTTGGTGGATTTTTCAGGAGTGTAGGAAAGGCTGTTCATTCAGTTCTCGGAGGACTCTTCAACACACTGTTTGGAGGAATGAGTTGGATTTCAAAAATTCTCATTGGAGTTCTCTTGGTATGGTTGGGCATTAGCGCACGTGACCACACACTAGCTGTCGCATTCATGTCTGTGGGGGGCATTCTGCTCTATCTGTCTACCTTGTCGGCAGCAGCATCTCTATCGGAAATAGGATGCAGTCTGGACATTAGC |
|  |  |
| BinJ/LAMV-prME | TTGCTGCTCGTTGGAGCAGGAGCGATGGCTGCTTCCATGTTCACAAGAGATGGGAAAGCACACTTGAATGTGTCGAGCAGTGATGTAGGAAAATGGCTACAGATAAAGACAGCAGTTGGAAATGGAACATGCATAGTGACAGCAACGGACGTTGGTTCATGGTGCGCAGATAATGTCAGGTACTTATGCCCTAGACTGGACAATGCAGCAGATCCCGATGACGTGGATTGTTGGTGCAACATAGTTTCAGTGTATGTTACATATGGCCGGTGCAAGAGGGAATCAAGCGGCCCAAGAAGAGGAAAGCGCTCAGTTGCACTTGCACCACATGGAACAGGAGACTTGCATACCGGGACAGCACCAATGTGGAAAGCACATTCTAGTGCACATCATTACTTGCAGCGAGTTGAACGATGGGCTCTAAGGAAGCCAGGATACCTGGCGGCCTTAGTGGCTATTGGATGGCTATTGGGCAAGACGAAGGCACAGAAAATTATCTACATAACGCTGCTTGTCCTCATCGGACCAGCATACAGTCTCCAGTGCGTTGACACCACTAACCGGGACTTCATTCAGGGAGTCAGCGGAGGCACTTGGGTTGACGTGGTACTTGACATAAACGGATGTGTGACGATCACAGCTGCCGATAAGCCCACAGTGGACTTTAAACTCGTTAAGCTCGAGATCACAAAAATGGCAGCAATCCGCTCTTATTGCCTCAAAGCTTCAACATCAGATGCTTCGTCGGTTAGTGGGTGCCCAGGAACAGGAGAGGTACATAACACCAAAGCCGAGGACACAGCATTCATATGTAAGAGCAGCAACCCAGACAGAGGATGGGGCAATGGATGTGGCCTTTTTGGAAAAGGATCCATGGAGACTTGTGCCAAGTTCACATGTGACAAGAAATTGGCAGGACATGTCATCAGCAGAGAGAACATTGAAGCTGAAGTTGACATTTCCATTCACGGACAGTCTGGAGCAGCAGATGACAACTCAACAAAGCGGAAAACCAGGAAGGAACTAGCAACGGTAACCATCACACCACAAACAGCTTCGGCTGAAGCTGACATGGGTGATTATGGAAAAATTGGGATCGATTGCTCCATGGACATAGGCATTGACTTCGAGCAAGTGGTCATCGCAGACGCTGGAGGACGGTATTGGATGCTTAGGAGGGACTGGTACCAAGATCTAGCGCTTCCCTGGACGGCCCCATCGGCAAATTTTTGGCATGATAGGGACAGGCTGATTGAGTTTGGAATACCACATGCCACGAAGCAAGGAGTGCATAACATCGGTGACCAGGAGGGAGCATTCCACACAGCAGCAGCGAAAGCACCAGCGGTGGAATACCATGAATCCAAAGTCAGACTGCCCACAGGTCTTCTTCGATGCAGAGTCAAGATGGGTAATATGAAGCTCAAAGGAACCACCTATTCACTTTGTACCGAGATGTTCACCTTTAGCAAAAGACCAGTGGATACCGGACATGGTACTGTGGTTTTCCAAGTCTCATACGCCGGCAACGATGCACCCTGCAAAATTCCTGTAGCCGTCACTGAAAAACCCAATGGTGAACCAACAGGACGACTAATCACTGCCCACCCAATCATATTGAAGAAAGATGACAGGGCAGTAGTAGAGGTTGAACCGCCATTTGGAAACAGTTACATTGAGATCGGCACAGCAACCAAAAAGATCACCGAAGTCTGGCACAAACCAGGAAGCTCTATAGGCAGTGCGTTTGTGTTGTCGTACAAAGGGCTCCAACGCCTTACGGTGATGGGGGAGCACGCATGGGACTTTGGATCAGTCGGAGGATTTTTCACTAGCCTGGGAAAAGCAGTGCACCACGTCTTCGGAAGCGTGTTCAGAACGCTCTTTGGAGGAATCAGTTGGATTGCCAAAATACTTATTGGAGGATTGCTGGTGTGGTTGGGGATAAGTGCGCGTGACCGCGTCTTAGCAACAACGTTCATCATAGTGGGCTCTATACTGCTGTATTTGGCAACCACAACTGTGGCCCTATCCCTATCGGAAATAGGATGCAGTCTGGACATTAGC |
|  |  |
| BinJ/NHUV-prME | GCTGCTCGTTGGAGCAGGAGCGATGGCTGTGACAGTGGGAACATTTGACAACAAGCCCCTTATTACGATCAAGGCAAATGAAGTTGGAAGGGCAATACACATCCCACAGCGGCATGGCAACTTGACCTGTGTGGTCAATGCCAATGATGTGGGTCAAATGTGTGATGACTCAATCACATACCTATGCCCTGACATTGACACAACGGATAGAGATGACATTGATTGCTGGTGCTCAGGAGGAGACGTGTATGTCAAGTATGGGAGATGCCATTCCGACAACAAGACAGCTCCCCACCGACGGTCACGGCGATCAGTGGCTCTTTCTCCCCACGGAGAGGGAGGCCTGAAAGTGAGAGGCAATAAATGGTTGGCGACAGATGCATCTGTTCTCCACCTGCAGAAAGTTGAAAGGTGGATGTTGAGCAATCCAGGATACGCTCTGGTGGCAGGAGTCCTTGGGGCTATGTTGGGAACAACAATGGTTCAGAAGGTTGTCATTACAGGGCTGCTGCTACTGGTCGCTCCGGCATACTCAACGCACTGCGTCAGATCTAACACACGTGACTTTGTCCAGGGAATCTCTGGAGGCACCTGGATTGATGTGGTGCTAGAGGGAGACGGGTGCGTGACCATCATGGCCGAAGGAAAACCTTCTGTTGACCTATCCTACATAAGGACAAGATTAACTAGCATGGCAAAAATCCGGACATACTGCTTGGAGGGAGCCATTTCAGACACCTCCACGGTGTCTCGCTGCCCATCCATGGGTGAGGCGTATAATGAAAAACGGAAAGACACATCCTATGTTTGCCACCAGGGTACATCGGGCCGTGGATGGGGAAGCGGATGTGGCTTGTTTGGCCAAGGCTCTCTAGACACCTGCGGAAAGTTTGCCTGTAGCAAAAAGATGATTGGGTACAAGGCTACTGTGGAGAACATTGAGCACAGCCTGAGATTAACTGTTCACGGCTCAGTGCATGGTGACAAGGTGGCTGATGAATCGCATTTGGCCACGCAGAAGTTAGGGAAAACATTCGCTATCACCCCAAAGGCCCCGGAAGTGGTCGTGGACCTCGGAGATTACGGGCAGGCCTCTGTTTCATGCCAAAAGGAAGCGGGATTGGACTATGAAAACACCATAGTTTTGGCTGTGGGAACCGAAGCGACCAACTCGAAAGTGTGGCTGGTTAACCAGCAGTGGTTTGAAGACATAGCATTGCCCTGGATCAGCGGGGAAGAAGATCTGTGGAGGAACAAAGAGCGCCTTGTTGAATTCCTGGGGCCTCACGCCACAAAGCAAGATATTGTTGTCCTAGGTGACCAGGAAGGAGCCATAATGCACGCTCTGGTGGGGACAACAAAAATATCCATTGCGTCCAACGCAGCCAGTGTGTTTGCTGGCCACTTGACGTGTCGAGTGAAAATGGAAAACCTAAAGATCAAAGGACTCACTTACCCGAATTGCGAAGGCACTTATTCTTTTGTCAAAGTGCCGTCGGACACCGGACATGGTACTATGATAACGGAAGTTAAGTCCACCACATCCAGTGTTCCTTGTAGACTAATTGTTGGGTTTGAAGACGCTAGTGGCAAAGTTCTTTCTGGGAGAATCATCACCACAAACCCGATCATAACGGCATCAGGAACCGGCGTTGTGGTCGAAGCAGAGGCTCCTTTTGGACCATCCACCTTCACAGTCGGCATGGGAACGCAGATGATCAAGTACCATTGGCATCGCAAGGGAAGCACCATTGGGGCTGCCCTAGCATCGGTCGTGACTGGAGCAAAACGGGTTGCCGTGATTGGCGACTCAGCCTGGGACTTCGGATCAGTTGGGGGAATCTTTAACTCCATGGGGAAAGCTGTTCACCAGATTTTTTCTGGTTTGTTCACAGCCCTTTTTGGGGGAATGAGCTGGGTGACAAAGGTCCTTGTTGGAGCATTGTTTGTGTGGATTGGAGCATCAGCGAAGAGCGAAAAGATTGCCATCTCCATGCTTGCCATTGGAGGAATCCTACTCTTCTTGGCCACCTCAGCGCACGCACTATCGGAAATAGGATGCAGTCTGGACATTAGC |
| BinJV/NOUV-prME | GCTGCTCGTTGGAGCAGGAGCGATGGCTGTGACGTTTACCACATTTGCAGGATTCCCACTGATGACAGTTGAACCTTCTGACATAGGAAAAGCCATAGCCGTACCGACACCGTCAGGTAACGTGACCTGTTACGTGCAAGCCAATACGGTTGGAGAGATGTGTGACCACAGTGTCACGTATCTATGCCCAAAGGTTGACGTGGAAAGAGAAGACGTGGACTGCTGGTGTCATGGAGGAGAAGCTTATGTTAGGTATGGAAAGTGCCGACGGGGAAAGAACCGAGCGCAACGTTCAAGAAGATCAGTGGGCATATCAGCCCACGGTAGCGGTGGTTTGGCTCATAAGAAAACAAGATGGATGTCAGTGGATGCCTCAATGGAACACTTCCAGAGAACTGAACGATGGGTTTTGAGAAACCCTGGATACGCTCTGATCGCAGGACTAATGGGATGGATGTTGGGTAGCAATCGCACTCAAAAGGTCATGTTCATTATCCTACTGCTGCTTGTCATTCCAGCGTATTCGATGAAATGCATAGGCGTGCAAAACCGTGACTTCATTACTGGAGTTAAAGGAACCACATGGGTTGATGTAGCCCTTGAAGCAGGTGGTTGTGTGACAATCACAGCCCAAGACAAGCCTACTATGGATATCATATTCACCCAATCCGTTGCCAAAAAACCAGCCCATGTCAGAAAAGTGTGTCTGGAAGCCAGCATAACGGAAATATCACACGTGGCGACCTGTCCAACTAATGGAGACGCTCACAACCCCAAAGCCAAAGACACTCTGTTCATGTGCAAGCGAGAATTGACCGACAGAGGATGGGGAAGCGGCTGTGCATTTTTTGCAAAAGGTAGTTTGGAAAGTTGCTGCAAGTTTGCTTGCAAAAAGAGCTACGACGCGAGCGTGATTACCAGAGAAAACATTGAGCATGCTCTCGAGGTGCACATACACACTGGAAAGGAATTGTACCATCATGGAAATGACTCACAGTTCGCGAAAGCCAAGACAGGGGCAGTTGTCAACTTTTCACCCAAAGCTTCCGAACAAACAGTTGATCTAGGAGACTATGGAACACTTGGACTAGTGTGTCGCGCAGAAGGTGGTGTGGAATATGAGACATCATATGTGTTTGGTAGGAAAGACAACAACAAGTACGCAAATGGGTGGCTGGCCAACCGAATGTGGGTTGATGATCTCCCATTGCCATGGACATCAGCCACTTCTGACCTTTGGCACAACAGAGAAGCACTCGTGGACTTTGGTGAGACTCATGCAGTGGAACGTTCAGTAGTGGTGCTAGGAGACCAAGAAGGCATGCTTATGAAAGCACTGGCCGGAGCCACCACACTGGACTTCTTTGAAGAAGCAGACACCGTGGGGGTGATCGCTGGTCATCTGACATGCCGCATCAAAGTGGAAAATCTGAAAATCAAAGGCGCCACCTACCCGGTATGCCCAAACACGGTTGTTCTTACCAAGGAACCTGTTGACACAGGCCACTCAACCGTGGTTGTGGAAGTGAAACTATCAACTTTCACCATGGCCTGCCGCCTGGCCGTAACTTTCACAGACGCGGCTGGAACCAAGATCACCGGAAGATTGATAACTGTCAATCCCATAATCACGGCTGCTGACGAGAAGGTCACGATAGAGATGGAGCCACCCTTTGGCGAATCCTTCATCGAAATTGGAGTGACAGCAGATTCAGTGAAGCATCACTGGAATCGGAAGGGTAGCTCAATAGGAGATGCATTCTACGCAACCTACAGAGGCGCCAGGAGGATGGCTGTCCTCGGGGATGCTGCCTGGGACTTCAACAGTATCGGCGGAGCTTTCAACTCCATTGGAAAAGGCGTTCACGCCATGTTTGGGAAAGTCTTCACAGTCCTATTCGGTGGGCTTAGCTGGATCTCGCAAATGGCCATCGGTGCATTGCTCATATGGTTGGGCGTTGGTGCTCGCAACAAGAGCATAGCAATAGGGATGATGGCTGTTGGCTCAATGCTCATATTCCTCTCCACTTCTGTTTCGGCACTATCGGAAATAGGATGCAGTCTGGACATTAGC |
