# Supplementary material for: Antigenic Characterization of New Lineage II Insect-Specific Flaviviruses in Australian Mosquitoes and Identification of Host Restriction Factors
Source: mSphere. 2020 Jun 17;5(3):e00095-20. doi: 10.1128/mSphere.00095-20 (PMC7300350; doi:10.1128/mSphere.00095-20)
Supplement: TABLE S4 [file mSphere.00095-20-st004.docx]

| **Table S4:** Fragments and primer sets used to generate BinJV CPER constructs | | | |
| --- | --- | --- | --- |
| **Fragment for CPER** | **Primer** | **Primer Sequence** | **Fragment Size (bp)** |
| 5’-UTR-E | F | AACGATCTGGTAAACAGTATATTTTGCGTG  TCCTATTTCCGATAGGGCACCCACGGTCAC | 2,490 |
|  | R |  |  |
| NS1-NS2B | F | GTGACCGTGGGTGCCCTATCGGAAATAGGA  CCACAACACAGTCCCCCGCTTGTTTGATTT | 2,166 |
|  | R |  |  |
| NS3-NS4B | F | AAATCAAACAAGCGGGGGACTGTGTTGTGG  GGTGGCCTGTAATCCCCTCCTAGGAACTCC | 3,108 |
|  | R |  |  |
| NS5 | F | GGAGTTCCTAGGAGGGGATTACAGGCCACC  GGAGTTCCTAGGAGGGGATTACAGGCCACC | 2,754 |
|  | R |  |  |
| 3’-UTR | F | GGCAATGTGATCTAAGGATCTACGAACGAG  TGCCATGCCGACCCAGATACTTGATGTTTCG | 479 |
|  | R |  |  |
| Linker | F | TGGATTGGGGATTGAGAAACATCAAGTATCTGGGTCGGCATGGCATCTCCACCTCCTCGC  GTGTTTTGAAACGCACACGCAAAATATACTGTTTACCAGATCGTTGCGGGCTGTATTTATAGGC | 1,019 |
|  | R |  |  |
