# Supplementary material for: Antigenic Characterization of New Lineage II Insect-Specific Flaviviruses in Australian Mosquitoes and Identification of Host Restriction Factors
Source: mSphere. 2020 Jun 17;5(3):e00095-20. doi: 10.1128/mSphere.00095-20 (PMC7300350; doi:10.1128/mSphere.00095-20)
Supplement: TABLE S5 [file mSphere.00095-20-st005.docx]

| **Table S5: Primer sets used to generate chimeric virus constructs*** | | |
| --- | --- | --- |
| **Fragment for CPER** | **Primer** | **Primer Sequence** |
| WNV_KUN_/BinJV-prME | KUNVC-BinJVprF | GCTGGCGTGGGAGCAGCCACTTTACGCACCG |
|  | KUNVC-BinJVprR | CGGTGCGTAAAGTGGCTGCTCCCACGCCAGC |
|  | BinE-KUN1F | GTGACCGTGGGTGCCGATACTGGATGTGCC |
|  | BinE-KUN1F | GGCACATCCAGTATCGGCACCCACGGTCAC |
| BinJ/CHAOV-prME | BinC-OH_F | GCTGCTCGTTGGAGCAGGAGCGATGGCT |
|  | BinC-OH_R | AGCCATCGCTCCTGCTCCAACGAGCAGC |
|  | BinNS1-OH_F | GAAATAGGATGCAGTCTGGACATTAGCCGAAAAG |
|  | BinNS1-OH_R | CTTTTCGGCTAATGTCCAGACTGCATCCTATTTC |
| BinJ/ILOV-prME | BinJVC-ILOVpr_F | GTTGGAGCAGGAGCGATGGCTACAACGGTGACCACCAGGGATGG |
|  | BinJVC-ILOVpr_R | CCATCCCTGGTGGTCACCGTTGTAGCCATCGCTCCTGCTCCAAC |
|  | ILOVE-BinJVNS1F | TTGTCGGCAGCAGCATCTCTATCGGAAATAGGATGC |
|  | ILOVE-BinJVNS1R | GCATCCTATTTCCGATAGAGATGCTGCTGCCGACAA |
| BinJ/LAMV-prME | BinJVC-LAMVpr_F | TTGCTGCTCGTTGGAGCAGGAGCGATGGCTGCTTCCATGTTCACAAGAGATGGGAAAGC |
|  | BinJVC-LAMVpr_R | GCTTTCCCATCTCTTGTGAACATGGAAGCAGCCATCGCTCCTGCTCCAACGAGCAGCAA |
|  | LAMVE-BinJVNS1F | CCACAACTGTGGCCCTATCCCTATCGGAAATAGGATGC |
|  | LAMVE-BinJVNS1R | GCATCCTATTTCCGATAGGGATAGGGCCACAGTTGTGG |
| BinJV/NHUV-prME | BinJVC-NHUVpr_F | CTCGTTGGAGCAGGAGCGATGGCTGTGACAGTGGGAACATTTGACAACAAGC |
|  | BinJVC-NHUVpr_R | GCAAATGTGGTAAACGTCACAGCCATCGCTCCTGCTCCAAC |
|  | NHUVE-BinJVNS1F | GCCACCUCAGCGCACGCACTATCGGAAATAGGATGC |
|  | NHUVE-BinJVNS1R | GCATCCTATTTCCGATAGTGCGTGCGCTGAGGTGGC |
| BinJV/NOUV-prME | BinJVC-NOUVpr_F | GTTGGAGCAGGAGCGATGGCTGTGACGTTTACCACATTTGC |
|  | BinJVC-NOUVpr_R | GCAAATGTGGTAAACGTCACAGCCATCGCTCCTGCTCCAAC |
|  | NOUVE-BinJVNS1F | TCCACTTCTGTTTCGGCACTATCGGAAATAGGATGC |
|  | BinJVC-NOUVpr_F | GTTGGAGCAGGAGCGATGGCTGTGACGTTTACCACATTTGC |
| *Primer sequences to generate BinJ/WNV_KUN_-prME have previously been published (6). | | |
